# Carabin deficiency in B cells increases BCR-TLR9 costimulation-induced autoimmunity

**DOI:** 10.1002/emmm.201201595

**Published:** 2012-10-29

**Authors:** Jean-Nicolas Schickel, Jean-Louis Pasquali, Anne Soley, Anne-Marie Knapp, Marion Decossas, Aurélie Kern, Jean-Daniel Fauny, Luc Marcellin, Anne-Sophie Korganow, Thierry Martin, Pauline Soulas-Sprauel

**Affiliations:** 1 CNRS UPR9021, IBMCStrasbourg, France; 2 UFR Médecine, Université de StrasbourgStrasbourg, France; 3 Department of Clinical Immunology, Hôpitaux Universitaires de StrasbourgStrasbourg, France; 4 EA4438, Groupe Borréliose de Lyme, UFR Sciences Pharmaceutiques and UFR Médecine, Université de StrasbourgStrasbourg, France; 5 Department of Anatomopathology, Hôpitaux Universitaires de StrasbourgStrasbourg, France; 6 UFR Sciences Pharmaceutiques, Université de StrasbourgIllkirch, France

**Keywords:** autoimmunity, B cells, Carabin, mouse models, systemic lupus erythematosus

## Abstract

The mechanisms behind flares of human autoimmune diseases in general, and of systemic lupus in particular, are poorly understood. The present scenario proposes that predisposing gene defects favour clinical flares under the influence of external stimuli. Here, we show that Carabin is low in B cells of (NZB × NZW) F1 mice (murine SLE model) long before the disease onset, and is low in B cells of lupus patients during the inactive phases of the disease. Using knock-out and B-cell-conditional knock-out murine models, we identify Carabin as a new negative regulator of B-cell function, whose deficiency in B cells speeds up early B-cell responses and makes the mice more susceptible to anti-dsDNA production and renal lupus flare after stimulation with a Toll-like Receptor 9 agonist, CpG-DNA. Finally, *in vitro* analysis of NFκB activation and Erk phosphorylation in TLR9- and B-cell receptor (BCR)-stimulated Carabin-deficient B cells strongly suggests how the internal defect synergizes with the external stimulus and proposes Carabin as a natural inhibitor of the potentially dangerous crosstalk between BCR and TLR9 pathways in self-reactive B cells.

## INTRODUCTION

Systemic lupus erythematosus (SLE), a prototype of human systemic autoimmune disease, is characterized by a wide variety of multi-organ damage (among which one of the hallmarks is glomerulonephritis), triggered by an autoantibody-mediated inflammation (Croker & Kimberly, 2005). The origin of SLE is generally attributed to a combination of a complex genetic influence (Flesher et al, 2010) and vaguely described environmental factors. In line with this theory, the majority of human SLE occurs in adults and is clinically characterized by a succession of flares interspersed with remission phases. This scenario strongly suggests the influence of flare-inducing external stimuli on a predisposing genetic background.

Several lines of evidence indicate that B cells are central to the disease process (Shlomchik & Madaio, 2003): 1) B cells produce the autoantibodies, some of which are clearly pathogenic forming immune complex deposits or destroying their target; 2) (NZB × NZW)F1 and MRL-Fas^lpr/lpr^ (murine models of human SLE) mice harbouring the *xid* mutation, which inactivates Btk and causes a blockade of B-cell development and B-cell responses, no longer develop a lupus phenotype, including autoantibodies and glomerulonephritis (Steinberg et al, 1982; 1983), as do (NZB × NZW)F1 mice having a very restricted IgM transgenic repertoire (Wellmann et al, 2001); 3) the disease can be transferred in mice by B cells since immunodeficient SCID (severe combined immunodeficiency) mice populated with pre-B cells of (NZB × NZW)F1 mice develop many of the characteristics of (NZB × NZW)F1 mice, suggesting that genetic defects responsible for the development of SLE disease in (NZB × NZW)F1 mice are present in their B cells (Reininger et al, 1996).

The study of SLE genetics has shown that the disease rarely occurs from a single mutation (except for deficiencies in the early components of complement cascade), but more commonly as a polygenic disease (Moser et al, 2009). On one hand, many polymorphisms of immune and non-immune genes (almost 30) have been described during the last 10 years, owing to large genome-wide association studies (GWAS) (Chung et al, 2011; Graham et al, 2008, 2009; Han et al, 2009; Hom et al, 2008; International Consortium for Systemic Lupus Erythematosus Genetics (SLEGEN) et al, 2008; Kozyrev et al, 2008; Yang et al, 2010, 2011) in lupus patients. They most likely constitute a set of predisposing SLE genes, but the consequences of these polymorphisms, in terms of protein levels or protein function, are generally unknown. Exceptions are BANK1, for which three variants have been associated to SLE and are supposed to lead to an altered B-cell activation threshold (Graham et al, 2009; Moser et al, 2009) and PTPN22, for which Zhang et al have recently developed a knock-in (KI) mouse line expressing the autoimmune disease-associated *PTPN22* variant (Pep619W). It is interesting to note that these mice show signs of lymphocyte hyperresponsiveness without developing pathogenic autoantibodies and signs of autoimmunity by their own (Zhang et al, 2011). On the other hand, and in parallel, genetically modified mice have been produced with clear functional consequences like a spontaneous autoimmune phenotype. For example, deficiencies of negative regulators of B lymphocytes induce spontaneous B-cell activation and spontaneous lupus phenotypes (Nitschke, 2005; Pritchard & Smith, 2003): 1) negative regulators of B-cell receptor (BCR) belonging to inhibitory co-receptors pathways [CD22 (O'Keefe et al, 1996; 1999; Otipoby et al, 1996; Poe et al, 2000), 9-*O*-acetyl sialic acid esterase or Siae (Cariappa et al, 2009), FcγRIIB (Bolland & Ravetch, 2000), PD-1 (Nishimura et al, 1999)], kinases phosphorylating BCR co-receptor ITIM motifs [Lyn (Hibbs et al, 1995)] and phosphatases recruited by the phosphorylated ITIMs [SHP1 (Pao et al, 2007)]; 2) B-cell-negative regulators of BCR-independent pathways like Act1 [TRAF3IP2 (Qian et al, 2004, 2008)] and the ubiquitin-modifying enzyme A20 [TNFAIP3 (Chu et al, 2011; Tavares et al, 2010)]. Although interesting, these data do not fit with the known discrete, not activated B-cell phenotype (normal expression of CD86 and CD40L) of lupus patients during the inactive phases of the disease, which precedes the flares. In addition, among the negative regulators of B cells described above, only two (A20 and FcγRIIB) have been shown to be candidate genes for human SLE (Moser et al, 2009). Altogether, these data suggest that SLE combines many minor susceptibility genes, each representing a single “hit” (for example the Pep619W allele) in a multi-hit and environment-dependent model for the development of SLE and of autoimmunity in general (Behrens, 2011).

In order to better undestand this model, it is important to identify gene defects, which can be responsible for an inducible, not a spontaneous, autoimmune disease phenotype, and to provide some mechanistic insights linking these defects to a flare-inducing environmental stimulus. In order to hunt down SLE predisposing gene abnormalities, and considering the important role of B cells in SLE, we started from a transcriptome analysis of B cells purified from SLE patients during latency phases of their disease and of B cells from young (NZW × NZB)F1 mice.

We identified a similar deficiency in *Carabin* gene expression in both human and murine lupus B cells. Carabin, alias TBC1D10C, was recently described as a negative regulator of T-cell function exhibiting a dual inhibitory activity on calcineurin (by its carboxy-terminal domain of interaction with calcineurin) and Ras (by its amino-terminus Ras/GAP domain) pathways. Knockdown of Carabin notably leads to a significant enhancement of IL-2 production by specific T cells after antigen stimulation (Pan et al, 2007). Considering the important molecular similarities of antigen receptor signaling in T and B cells, including the role of Ras and Calcineurin pathways in BCR signaling, we decided to evaluate the role of Carabin in B cells, which is currently unknown, and to look for signs of autoimmunity in Carabin-deficient mice. Using knock-out and B-cell-conditional knock-out murine models, we show that Carabin is a new negative regulator of the Ras/Erk pathway in B cell. The phenotype of Carabin-deficient B cells in non-autoimmune prone mice is subtle: although characterized by an acceleration of early B-cell response after immunization, Carabin knock-out (KO) mice do not present any spontaneous B-cell activation, nor spontaneous production of autoantibodies. However, when Carabin-deficient mice are stimulated with a Toll-like Receptor 9 (TLR9) agonist (CpG-DNA), thereby mimicking a viral infection, we observe the production of anti-dsDNA antibodies and a lupus-like glomerulonephritis with immune deposits in a subgroup of mice. Finally, our *in vitro* data give some mechanistic insights into these results, proposing Carabin as a new negative regulator of TLR9/BCR costimulation in self-reactive B cells.

## RESULTS

### Carabin expression is low in lupus B cells in the quiescent phase of the disease

**Figure 1:**
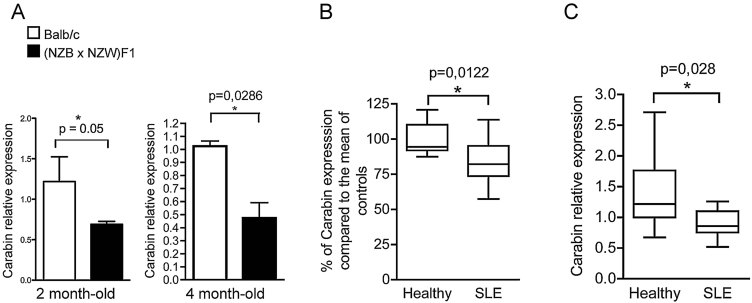
Carabin is underexpressed in (NZB × NZW)F1 and in lupus patient B cells Quantitative real-time RT-PCR analysis of *Carabin* mRNA expression in splenic mature B cells purified from 2 month-old Balb/c (*n* = 3) and (NZB × NZW)F1 (*n* = 4) mice (left), and from 4 month-old Balb/c (*n* = 4) and (NZB × NZW)F1 (*n* = 4) mice (right). Each sample was normalized to the endogenous control *Hprt1*. (errors bars, standard deviation). *p < 0.05, Mann & Whitney test.*Carabin* mRNA expression levels in transcriptoma analysis of purified B cells from 17 SLE patients, compared to 9 healthy age and sex-matched controls.Quantitative real-time RT-PCR analysis of *Carabin* mRNA expression in B cells purified from the blood of 10 lupus patients (SLE) compared to 10 healthy individuals. Each sample was normalized to the endogenous control *Hprt1*.

In a transcriptome analysis of purified splenic B cells from 4-month-old (NZB × NZW)F1 mice (8–10 weeks before the occurrence of the disease), *Carabin* mRNA expression was lower than in control mice (*p* < 0.05, unpublished observation). A 50% reduction of *Carabin* expression was confirmed by real-time quantitative RT-PCR in B cells from 4 month-old as well as in 2 month-old (NZB × NZW)F1 mice (43% reduction), a long time before the appearance of autoantibodies, B-cell hyperactivation and disease in this SLE model (Fig 1A). We also performed a pangenomic transcriptome analysis (Affymetrix GeneChip human genome U133 plus 2.0) of purified B cells from 17 patients with SLE in quiescent phase [SLE disease activity score (Selena SLEDAI) less than 4] compared to B cells from age- and sex-matched controls (Garaud et al, 2011). We studied patients with inactive disease and with minimum treatment (less than 10 mg/day of prednisone and no immunosuppressive treatment) in order to avoid background due to non-specific B-cell activation that occurs during flares of the disease. Indeed, the concomitant immunophenotyping of these patients B cells did not show any sign of activation (normal expression of CD86) (Korganow et al, 2010). These data have been deposited in NCBI's Gene Expression Omnibus (Edgar et al, 2002) and are accessible through GEO Series accession number GSE30153 (http://www.ncbi.nlm.nih.gov/geo/query/acc.cgi?acc=GSE30153). Most interestingly, *Carabin* is significantly underexpressed in SLE patients (*p* = 0.01) (Fig 1B). In addition, we confirmed by real-time quantitative RT-PCR in a new cohort of 10 SLE patients the low level of Carabin expression in purified B cells compared to healthy controls (ranging from 30 to 60% reduction, Fig 1C).

During our transcriptome analysis of SLE B cells, Pan et al. identified Carabin as a negative regulator of T-cell function (Pan et al, 2007). We thus decided to further investigate Carabin function in B cells.

### Carabin is a new negative regulator of the Erk pathway in B cells

**Figure 2:**
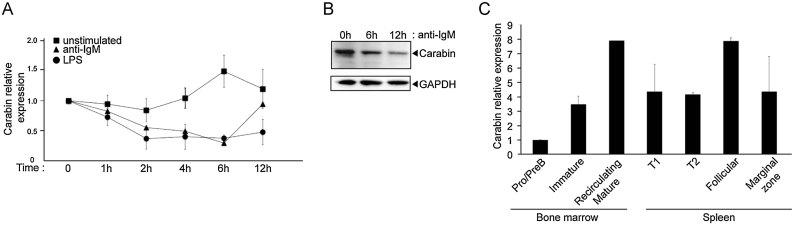
Carabin expression is tightly regulated during B-cell maturation and activation Data correspond to three independent experiments. **A,B.** Purified splenic mature B cells from C57/BL6 mice were stimulated with anti-IgM (10 µg/ml), LPS (10 µg/ml), or left unstimulated for the indicated time. Carabin expression was evaluated by (A) quantitative real-time RT-PCR or (**B**) Western blot. (**A**, errors bars, standard deviation)**C.** Quantitative real-time RT-PCR analysis of *Carabin* expression in FACS-sorted B-cell subsets from C57/BL6 mice: Pro/PreB (B220^+^IgM^*−*^); Immature (B220^med^IgM^+^); recirculating mature (B220^high^IgM^+^); T1 (IgM^+^CD23^*−*^CD21^*−*^); T2 (IgM^+^CD23^+^CD21^high^); follicular (IgM^+^CD23^+^CD21^low^); marginal zone (IgM^+^CD23^*−*^CD21^high^). Samples were normalized to the endogenous control *Hprt1*. (errors bars, standard deviation).

Since the role of Carabin in B-cell function was unknown, we analysed the variation of Carabin expression in normal murine B cells after activation and during B-cell development. BCR or LPS activation leads to a fast decrease of Carabin expression (Fig 2A and B). In addition, Carabin expression was tightly regulated during B-cell maturation in normal mice, with a gradual increase from bone marrow pro/preB cells to splenic mature follicular B cells (Fig 2C). In conclusion, the high expression of Carabin in mature B cells could be indicative of a more important role of Carabin in mature B-cell function.

**Figure 3:**
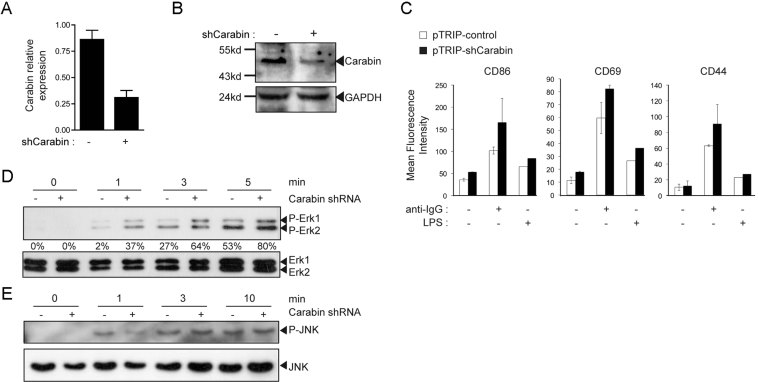
Carabin underexpression increases Erk1/2 phosphorylation after stimulation of A20 B cells Data correspond to three independent experiments. A20 cells were transduced with lentiviral constructs containing no shRNA (pTRIP-control, −) or an shRNA targeting *Carabin* (pTRIP-shCarabin, +) and GFP^+^, transduced cells were sorted. Carabin expression was determined by quantitative real-time RT-PCR. Each sample was normalized to the endogenous control 18S. Bars represent the level of *Carabin* transcript expression in transduced GFP^+^ A20 cells relative to non-transduced A20 cells. (errors bars, standard deviation).Immunoblot analysis of Carabin expression in A20 B cells after transduction as in **A**. GAPDH was used as loading control.Flow cytometry analysis of activation markers on GFP^+^ A20 B cells, transduced and sorted as in **A**, then stimulated with an anti-IgG antibody (10 µg/ml) or with LPS (10 µg/ml) for 24 h. (errors bars, standard deviation).A20 B cells transduced and sorted as in **A**, then stimulated with an anti-IgG antibody (10 µg/ml) for 1, 3 or 5 min. Cell lysates were analysed by Western blot using anti-phospho Erk1/2 antibody. Erk1/2 was used as a loading control. The percentages of phospho-Erk1/2 were normalized to the total Erk1/2 proteins in the corresponding lane, and then to unstimulated cells (time 0, 0%). Notation: +, cells stably expressing *Carabin* specific shRNA; −, control cells.A20 B cells were treated as in **D**. Cell lysates were analysed by Western blot using anti-phospho JNK antibody. JNK was used as a loading control. Notation: +, cells stably expressing *Carabin* specific shRNA; −, control cells.

To clarify the function of Carabin in B cells, we first analysed the phenotype of Carabin knock-down (KD) in a IgG^+^, A20 B-cell line transduced with a pTRIP lentivirus allowing for the coexpression of a *Carabin*-specific shRNA and GFP reporter gene. pTRIP-shCarabin-transduced A20 B cells showed a 70% decrease of Carabin expression compared to pTRIP-control-transduced A20 B cells as assessed by quantitative real-time RT-PCR (Fig 3A). The reduced expression of Carabin was further confirmed by Western Blot analysis (Fig 3B). When compared to control A20 B cells, Carabin KD-A20 B cells displayed a modest increase in the expression of CD86 and CD69 activation markers before and after stimulation with LPS or anti-IgG antibody (Fig 3C). Because Carabin has been shown in T cells to inhibit Ras pathway and Erk1/2 phosphorylation, we evaluated the effects of Carabin KD on the Ras pathway in A20 B cells. Interestingly, Carabin KD accelerated Erk1/2 phosphorylation in BCR-stimulated B cells (Fig 3D). This effect was specific for the BCR pathway, because LPS stimulation did not lead to a faster Erk1/2 phosphorylation in Carabin KD-A20 B cells compared to control B cells. To test the specificity of Carabin for the Ras MAP kinase signaling pathway in B cells, we analysed the activation of another related member of the MAP kinase superfamily, c-Jun N-terminal kinase, which is not targeted by Ras. JNK phosphorylation was not affected by Carabin KD in A20 B cells (Fig 3E). In conclusion, Carabin is a negative regulator of Ras/Erk pathway in B cells, as described for T cells (Pan et al, 2007).

### Carabin is not involved in B- and T-cell development and in the basal secretion of immunoglobulins

In order to fully analyse Carabin function *in vivo*, we generated Carabin KO and conditional KO mice (see Materials and Methods Section, and Supporting Information Fig 1). *Carabin*^−/−^ mice were obtained with a Mendelian frequency and developed normally. The development of B and T cells was extensively studied in these mice. Concerning T-cell development, total cellularity of thymus, spleen and lymph nodes and percentages of developing thymocytes and of mature CD4^+^ and CD8^+^ T cells were comparable in *Carabin*^−/−^ and control littermate *Carabin* ^*+/+*^ mice (Supporting Information Table 1), thus confirming in a physiological model that Carabin does not influence T-cell maturation as proposed by Pan et al in their Carabin KD hematopoietic stem cell transfer model (Pan et al, 2007). Similarly, there was no statistical difference in the absolute numbers and proportions of the different sub-populations of B cells in primary and secondary lymphoid organs in *Carabin*^−/−^ and control mice (Supporting Information Table 1). In addition, there was no noticeable difference in the structures of the spleens and lymph nodes. Finally, at baseline, the secretion of serum IgM, IgG and IgG subtypes was not statistically different between the two groups of mice (Supporting Information Fig 2). Thus, Carabin is not involved in B- and T-cell development and in the basal secretion of immunoglobulins.

### Increased response of Carabin^*−*/*−*^ B and T cells *in vitro*

**Figure 4:**
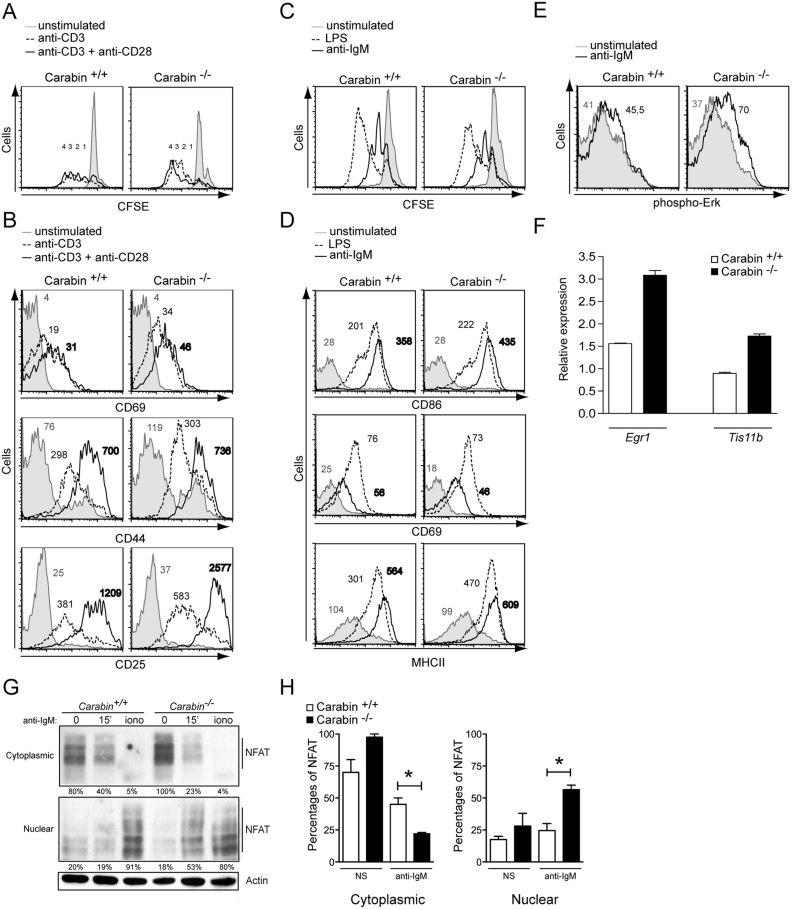
Increased response of Carabin-deficient T and B Cells **A,B.** Flow cytometry analysis of (**A**) dilution of CFSE-labeled and (**B**) cell surface expression of CD69, CD44 and CD25 on *Carabin*^*+/+*^ and *Carabin*^−/−^ CD4^+^ T cells after stimulation for 72 h with anti-CD3 antibody (2 µg/ml) (dashed line), anti-CD3^+^anti-CD28 antibodies (2 µg/ml each) (solid line), or medium alone (shaded gray). Data in **A** correspond to three independent experiments. The corresponding statistical to B analysis is represented in Supporting Information Fig 3A.**C,D.** Flow cytometry analysis of (**C**) dilution of CFSE-labeled and (**D**) cell surface expression of CD86, CD69 and MHCII on *Carabin*^*+/+*^ and *Carabin*^−/−^ CD19^+^ B cells after stimulation for 72 h with LPS (10 µg/ml) (dashed line), anti-IgM antibody (10 µg/ml) (solid line), or medium alone (shaded gray). Data in **C** correspond to three independent experiments. The corresponding statistical analysis to **D** is represented in Supporting Information Fig 3C.**E.** Flow cytometry analysis of Erk phosphorylation in *Carabin*^*+/+*^ and *Carabin*^−/−^ B220^+^ B cells after stimulation for 10 min with anti-IgM antibody (10 µg/ml) (solid line), or with medium alone (shaded gray). Numbers indicate Mean Fluorescence Intensity. The corresponding statistical analysis is represented in Supporting Information Fig 3D.**F.** Purified splenic mature B cells were stimulated for 1 h with anti-IgM antibody (0.5 µg/ml), or medium alone. Egr1 and TIS11b expression was determined by quantitative real-time RT-PCR. Each sample was normalized to the endogenous control Hprt1. Bars represent the level of Egr1 and TIS11b transcript expression in *Carabin*^*+/+*^ and *Carabin*^−/−^ anti-IgM stimulated B cells relative to unstimulated B cells.**G,H.** Immunoblot analysis of NFAT nuclear translocation in *Carabin*^*+/+*^ and *Carabin*^−/−^ splenic purified (CD43-negative) B cells after stimulation for 15 min with anti-IgM antibody (5 µg/ml), ionomycin (1 µM) or medium alone. Actin was used as loading control. The percentages indicate the amount of NFAT, which has been normalized to the actin's amount, and are represented in **H** for three independent experiments. *p < 0.05, Mann & Whitney test.

To further study the role of Carabin in B- and T-cell function, we analysed the response of Carabin-deficient B and T cells *in vitro* and confirmed and completed Pan's description of Carabin deficiency in T cells. *Carabin*^−/−^ T cells displayed an increased proliferative response (CFSE assay) after stimulation with anti-CD3 or anti-CD3/anti-CD28 antibodies (Fig 4A), an increased spontaneous expression of CD25 and CD44 activation markers at baseline, and an increased expression of CD69 and CD25 after stimulation with anti-CD3 or anti-CD3/anti-CD28 antibodies (Fig 4B and Supporting Information Fig 3A). The phosphorylation of Erk was also enhanced in *Carabin*^−/−^ T cells before and after stimulation with anti-CD3 or anti-CD3/anti-CD28 antibodies (Supporting Information Figs 3B and 4) in concordance with the data obtained by Pan et al showing that Erk phosphorylation was delayed when N-terminal Ras GAP domain of Carabin was overexpressed in PMA/ionomycin-activated human Jurkat T cells (Pan et al, 2007). On the contrary, considering the B cells of *Carabin*^−/−^ mice, we made the following observations: 1) the proliferation of B cells (Fig 4C) and the increase of expression of activation markers (CD86, CD69, MHCII) on B cells (Fig 4D and Supporting Information Fig 3C) in response to BCR-dependent (anti-IgM) or BCR-independent (LPS) stimulation is not different in *Carabin*^−/−^ and in control *Carabin*^*+/+*^ mice; 2) there was no difference in Ig production between *Carabin*^−/−^ and ^*+/+*^ mice after stimulation of splenic cells with LPS or LPS plus IL4 *in vitro* (Supporting Information Fig 5); 3) *Carabin*^−/−^ B cells showed an increase of Erk phosphorylation compared to *Carabin*^*+/+*^ B cells after BCR activation with anti-IgM antibody (Fig 4E and Supporting Information Fig 3D) confirming the results obtained in Carabin KD-A20 B cells (Fig 3D). This increase or Erk phosphorylation led to a higher induction of Egr1 and TIS11b (Fig 4F), both known to be induced in B cells after BCR stimulation in a Erk-dependent manner (Glynne et al, 2000). Because Carabin has a dual inhibitory activity on Ras and calcineurin pathway in T cells (Pan et al, 2007), we have also analysed the nuclear factor of activated T cells (NFAT) nuclear translocation in *Carabin*^−/−^ B cells after BCR stimulation and have shown a decrease of cytoplasmic NFAT and an increase of nuclear NFAT in *Carabin*^−/−^ B cells compared to *Carabin*^*+/+*^ B cells (Fig 4G and H). However, Carabin does not seem to play an inhibitory role on more usptream signals such as calcium influx (Supporting Information Fig 6A and B). To conclude, stimulation-induced increase of Erk activation and NFAT nuclear translocation appears to be a common consequence of Carabin deficiency in T and B cells. But in contrast to T cells, Carabin deficiency in B cells is not associated with a spontaneous activation status.

### Carabin deficiency accelerates early B-cell responses *in vivo*

**Figure 5:**
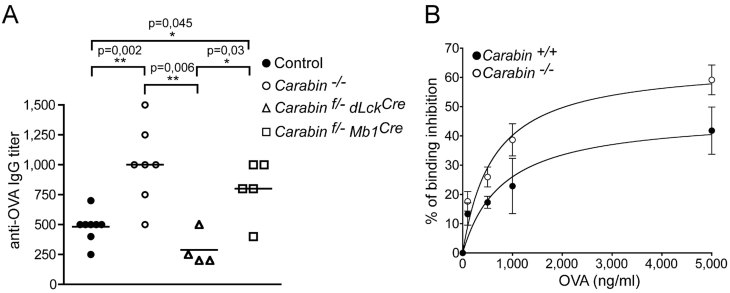
Increased early antigen-specific B-cell response in Carabin KO and Carabin conditional KO mice Six- to eight-week-old mice of the indicated genotype were injected intraperitoneally with 100 µg OVA in complete Freund's adjuvant and bled 7 days after injection. Anti-OVA IgG titers were determined by ELISA. Each point represents the result for one animal. The results of two-tailed Mann–Whitney test are indicated. Because the results obtained in *Carabin*^+/+^ and in *Carabin*^f/*−*^ Cre-negative mice were not different, these animals were pooled in the same “control” group.The relative affinities of anti-OVA IgG in the *Carabin*^−/−^ (*n* = 7) and control mice (*n* = 4) were determined by a competitive inhibition ELISA assay, using increasing concentrations of soluble OVA in the ELISA assay presented in **A**. (errors bars, standard deviation). *p < 0.05, **p < 0.005, Mann & Whitney test.

Despite this subtle phenotype, B-cell-specific Carabin-deficient mice have accelerated antigen-specific B-cell responses *in vivo*, indicating a physiological role of Carabin in B cells. Indeed, after exposure to a T-cell-dependent antigen (ovalbumin, OVA), Carabin deficiency speeds up the production of anti-OVA IgM (Supporting Information Fig 7) and IgG (Fig 5A) 7 days after immunization, although the difference was statistically significant only for IgG. In addition, this increase in anti-OVA IgG was not observed in *dLck-Cre* Carabin-conditional KO mice, but was still observed in *Mb1-Cre* Carabin-conditional KO mice (Fig 5A). These results indicate that the early B-cell response to T-cell-dependent antigen in Carabin KO mice is linked to a deficiency of Carabin in B cells, but not in T cells. This effect is transient since at day 14, the levels of anti-OVA IgG were comparable in *Carabin*^−/−^ and control mice. In order to test if this early B-cell switch was also associated with a change in the affinity of the antigen-specific IgG antibodies, we compared the relative affinities of anti-OVA IgG in *Carabin*^*+/+*^ and *Carabin*^−/−^ mice at day 7 after immunization in a competitive inhibition ELISA assay. Although the difference was not statistically significant, the affinity of antigen-specific IgG tended to be slightly higher in *Carabin*^−/−^ mice (Fig 5B). Finally, at day 7 after immunization, the percentages of OVA-specific B cells (B220^+^/OVA^+^) and of germinal centre B cells (CD95^+^/GL7^+^) in OVA-specific population were similar in the spleen or lymph nodes of *Carabin*^−/−^ and control mice (Supporting Information Fig 8A and B), indicating that there was no drastic modification in the germinal centre kinetics of *Carabin*^*−*/*−*^ OVA-specific B cells compared to *Carabin*^*+/+*^ OVA-specific B cells. Similarly, the T-cell-independent IgG B-cell response in the absence of Carabin [immunization with 4-hydroxy-3-nitrophenylacetyl (NP)-LPS] was enhanced 7 days after immunization (Supporting Information Fig 9A and B). We also investigated the B-cell response in Carabin-deficient mice in an experimental infection. *Carabin*^*−*/*−*^ and control mice were infected with *Borrelia burgdorferi* (Bb) producing a chronic-systemic infection. The anti-Bb specific IgG response was more pronounced in the absence of Carabin from day 14 to day 22 (Supporting Information Fig 10). In conclusion, the early B-cell response to both T-dependent and T-independent antigens is transiently increased in the absence of Carabin. In addition, the B-cell response is also enhanced in the absence of Carabin after an infectious event. Altogether, these results could be linked to the acceleration of Erk phosphorylation and NFAT nuclear translocation that we observed *in vitro* after BCR-dependent stimulation of Carabin KD and KO B cells.

### A subgroup of *Carabin*^*−*/*−*^ and B-cell-specific *Carabin*-deficient mice develop signs of autoimmunity after stimulation with the TLR9 agonist CpG-DNA

In order to check if the enhanced B-cell response in the absence of Carabin could lead to autoimmunity, we analysed the development of autoantibodies and autoimmune signs in *Carabin*-deficient mice. *Carabin*^*−*/*−*^ mice did not spontaneously produce antinuclear autoantibodies and in particular anti-dsDNA autoantibodies, nor did they develop any symptoms of autoimmunity (including glomerulonephritis) even at older ages (12–14 month-old).

**Figure 6:**
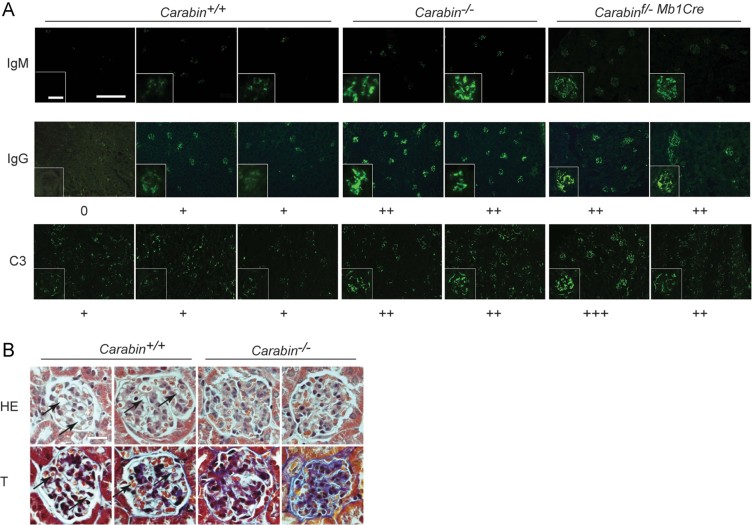
Autoimmunity in CpG-DNA treated *Carabin^−/−^* and B-cell specific *Carabin^−/−^* mice Eight to ten-week-old mice of the indicated genotypes were treated with 40 µg of CpG-DNA intraperitoneally every other day for 2 weeks (see also Table 1). Immunofluorescent analyses of glomerular deposition of Igs of the indicated isotypes and C3 after CpG-DNA treatment. Mice were analyzed 6 weeks after start of treatment. The score (0, +, ++, +++) of IgG and C3 deposits in renal glomeruli is indicated. Scale bars: 200 and 20 µm.Hematoxylin and eosin (HE) and trichrome (T) sections of *Carabin*^+/+^ and *Carabin*^*−*/*−*^ mice. The arrows show the glomerular capillaries, whose diameter is reduced in *Carabin*^−/−^ mice due to the increase of mesangium cellularity. Scale bars: 200 µm.

Since Carabin expression is low in lupus B cells in mice and men and despite the subtle phenotype we observed in deficient mice, we asked if its deficiency was able to sensitize naturally occuring autoreactive B cells and provoke autoimmunity. Overt autoimmunity is likely a multistep process that can involve the escape of autoreactive B cells from negative selection, but also abnormal B-cell responses after activation of the innate immune system and particularly of TLR pathways (Baccala et al, 2007; Marshak-Rothstein, 2006; Peng, 2005; Soulas, 2005). Thus, we immunized *Carabin*^−/−^ and control mice with CpG-DNA for the following reasons: 1) CpG-DNA is a TLR9 agonist, which mimics an infectious agent; 2) TLR9 is a known B-cell sensor of dsDNA viruses like EBV and Parvovirus B19, which are linked to SLE flares (McClain et al, 2005); 3) the possible interplay between self-antigen BCR activation and TLR activation (Leadbetter et al, 2002); and finally 4) CpG-DNA is known to induce type I interferon production, which is a key factor for SLE pathogenesis (Baccala et al, 2007; Theofilopoulos et al, 2005). Anti-dsDNA production and deposition of IgG and C3 in renal glomeruli were analysed in CpG-DNA-treated *Carabin*^*−*/*−*^, B-cell-specific *Carabin*^*−*/*−*^ and *Carabin*^+/+^ mice. CpG-DNA treatment induced a higher production of anti-dsDNA IgG at D14, D28 and D49 (with a titer higher than 40) in *Carabin*^*−*/*−*^ and B-cell-specific *Carabin*^*−*/*−*^ compared to control mice (Table 1). The scenario was the same (and statistically significant when comparing control mice and B-cell-specific Carabin-deficient treated mice) if we considered the highest score of IgG and C3 deposits in renal glomeruli (Table 1, illustrated in Fig 6A). In some mice with high amounts of renal IgG deposits, classical histological analysis was very similar to mesangial type II lupus glomerulonephritis with a clear increase of mesangium cellularity [ISN/RPS classification (Weening et al, 2004)] (Fig 6B). In addition, there was a good correlation between IgG and C3 deposits (Fig 6A and Table 1). It seems that CpG-DNA immunization directly acts on spontaneously occurring anti-DNA B cells because anti-thyroglobulin and anti-actin autoantibodies did not increase in anti-dsDNA-positive *Carabin*^−/−^ mice compared to control mice (Supporting Information Fig 11A and B). This strongly suggests that the higher production of anti-dsDNA in Carabin-deficient mice is more the consequence of an anti-dsDNA BCR-specific pathway than of a non-specific polyclonal activation. Noteworthy, NP-LPS (TLR4 ligand) immunization led to low level production of various autoantibodies (anti-dsDNA and anti-thyroglobulin) in both groups of mice, but without any significant difference between *Carabin*^−/−^ and^*+/+*^ mice (Supporting Information Fig 9C and D). In conclusion, although the phenotype is partial, the treatment of *Carabin*^*−*/*−*^ and B-cell-specific *Carabin*^*−*/*−*^ mice with TLR9 agonist makes them more susceptible to signs of renal lupus flares.

**Table 1:** Production of anti-dsDNA and deposition of IgG in renal glomeruli in CpG-DNA treated *Carabin*^−/−^ and B-cell specific *Carabin*^−/−^ mice

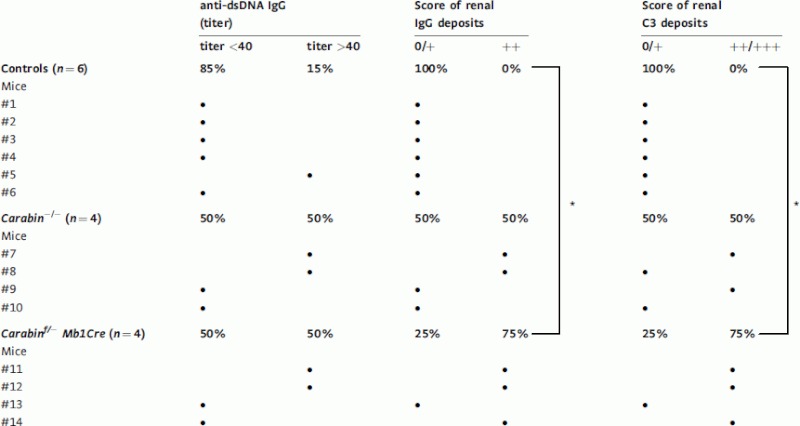

Eight to ten-week-old mice of the indicated genotypes were treated with 40 µg of CpG-DNA intraperitoneally every other day for 2 weeks. Left: serum was collected before treatment (day 0), and at day 14, 28, 49 after the first injection. Anti-dsDNA IgG titers were determined by ELISA. The percentages of mice presenting an anti-dsDNA titer inferior to 40 and superior to 40 are represented in each group. Results obtained at day 14, 28 and 49 were identical. Middle and Right: immunofluorescent analyses of glomerular deposition of IgG (middle) and C3 (Right) after CpG-DNA treatment. Mice were analyzed 6 weeks after start of treatment. The intensity of renal IgG and C3 deposits was scored. The percentages of mice presenting a score of 0/+ and a score of ++ (for IgG) or ++/+++ (for C3) are represented in each group. ^*^p < 0.05, Fisher exact's test.

### Carabin prevents dangerous TLR9-induced Erk activation in BCR-stimulated B cells

**Figure 7:**
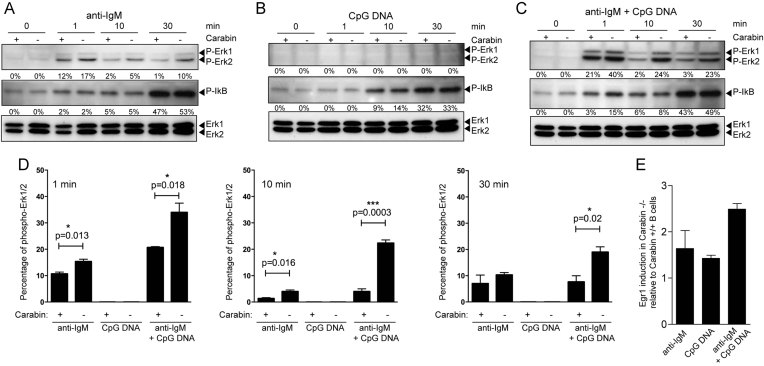
Carabin prevents TLR9-induced Erk activation in BCR stimulated B cells Data correspond to three independent experiments. *p < 0.05, ***p < 0.0005, Mann & Whitney test. **A-D.** Purified splenic mature B cells from *Carabin*^*+/+*^ and *Carabin*^−/−^ mice were stimulated with anti-IgM (10 µg/ml) (**A**), CpG-DNA (1 µg/ml) (**B**), or anti-IgM (10 µg/ml) plus CpG-DNA (1 µg/ml) (**C**) for the indicated time. Cell lysates were analysed by Western blot using anti-phospho Erk1/2 and anti-phospho IκB-α antibody. Erk1/2 was used as a loading control. The percentages of phospho-Erk1/2 and phospho-IκB-α were normalized to the total Erk1/2 proteins in the corresponding lane, and then to unstimulated cells (time 0, 0%), and represented in (**D**) for phospho-Erk1/2.**E.** Purified splenic mature B cells were stimulated for 1 h with anti-IgM antibody (5 µg/ml), CpG-DNA (1 µg/ml), anti-IgM (5 µg/ml) plus CpG-DNA (1 µg/ml) or medium alone. Egr1 expression was then determined by quantitative real-time RT-PCR. Each sample was normalized to the endogenous control Hprt1. Egr1 induction in *Carabin*^*+/+*^ and *Carabin*^−/−^ B cells relative to unstimulated B cells was calculated. Bars represent the ratio of Egr1 induction in *Carabin*^−/−^ B cells relative to *Carabin*^*+/+*^ B cells.

In order to obtain mechanistic insight into the previous results, we investigated the BCR and TLR9 pathways, which both could be involved. Carabin-sufficient or -deficient purified B cells were *in vitro* cultured after BCR stimulation (anti-IgM) and/or TLR9 stimulation (CpG-DNA) before Erk phosphorylation and IκB-α activation were measured. Carabin-deficient B cells presented an increase and an acceleration of Erk phosphorylation after anti-IgM stimulation compared to control B cells (Fig 7A and D) confirming the data obtained by flow cytometry (Fig 4E) without any modification of NF-κB activation (Fig 7A). NF-κB activation following CpG-DNA stimulation alone was not modified by the lack of Carabin in B cells, and under our experimental conditions, CpG-DNA did not induce any activation of the Erk pathway in Carabin-deficient or control B cells (Fig 7B and D). Interestingly, CpG-DNA induced not only an increased but also a sustained Erk phosphorylation in BCR-stimulated, Carabin-deficient B cells (Fig 7C and D). The expression of the Erk target gene *Egr1* was also increased by the costimulation (Fig 7E). Because Egr1 has been shown to regulate activation-dependent CD44 transcription in B cells (Dinkel et al, 1997; Maltzman et al, 1996), we analysed CD44 expression after BCR and TLR9 costimulation and showed that the increase of CD44 expression after costimulation is higher in B cells from *Carabin*^−/−^ mice compared to *Carabin*^*+/+*^ mice (Supporting Information Fig 12A and B). A connection between TLR9 activation and Ras activation has already been described in macrophages (Xu et al, 2003). Taken together, these results strongly suggest that this Ras-TLR9 crosstalk could also exist in B cells and that Carabin, by negatively regulating Ras activation, prevents excessive BCR-dependent B-cell activation, which could be provoked by TLR9 co-stimulation (Supporting Information Fig 13).

## DISCUSSION

Carabin was initially identified by transcriptome analysis of murine and human SLE B cells during clinically inactive phases of the disease. The low level of *Carabin* mRNAs in these cells incited us to define the function of this protein in B cells through a functional genomic approach and to investigate the way by which carabin deficiency could lead to Lupus flares.

Here, we show that Carabin is a negative regulator of B cells. The KD or KO of Carabin in B cells triggers an acceleration of Erk phosphorylation and an accelerated response to both T-dependent and T-independent antigens. Our results further confirm in murine T cells the phenotype of Carabin deficiency by showing an increased phospho-Erk expression (Pan et al, 2007). In addition, we show an increased expression of activation markers in stimulated *Carabin* KO T cells, which was associated with an increased proliferation. However, it seems that the consequences of Carabin deficiency in T and B cells are noticeably different, although both share the common acceleration of the Erk pathway after antigen-specific stimulation. This suggests that the Erk pathways display some differences in B and T cells, either in the molecular partners or in the downstream targets of Erk.

Though Carabin is another negative regulator of B cells, it takes a special place among the others: 1) Carabin KO mice do not present any defect in B-cell development. This contrasts with other studies on negative regulators of B cells, which indicate that BCR signal strength may control the development of immature B cells in the different mature populations (follicular, marginal zone, B1) (Pillai & Cariappa, 2009). A possibility could be that the effect of Carabin deficiency on BCR signaling is not strong enough to induce detectable differences on these B-cell subpopulations. 2) The phenotype of Carabin-deficient B cells is quite moderate without any alteration in B cells or spontaneous production of autoantibodies. This subtle phenotype is unique compared to the phenotype of mice deficient for other negative regulators since a defect in CD22 (O'Keefe et al, 1996; 1999; Otipoby et al, 1996; Poe et al, 2000), Lyn (Chan et al, 1998; Hibbs et al, 1995) or SHP1 (Pao et al, 2007), belonging to the same pathway, leads to a common phenotype characterized by increased Ca^2+^ signaling in B cells after BCR stimulation, a decrease of MZ and mature B-cell populations in spleen and spontaneous production of antinuclear autoantibodies associated with the development of glomerulonephritis. Similarly, a defect in FcγRIIB/SHIP pathway triggers an increased Ca^2+^ response, an increased activation of Erk2 and Akt pathways and also (for FcγRIIB) the spontaneous production of antinuclear autoantibodies associated with the development of glomerulonephritis (Bolland & Ravetch, 2000; Helgason et al, 2000; Liu et al, 1998; Takai et al, 1996). Finally, a deficiency of A20 leads to an hyperactivation of B cells after BCR, LPS or CD40 activation and to the spontaneous production of autoantibodies but, similar to Carabin deficiency, an A20 defect is associated with the development of glomerulonephritis after TLR9 stimulation (Chu et al, 2011; Tavares et al, 2010). However, and most interestingly, the subtle phenotype of Carabin-deficient mice and the fact that they do not develop autoimmune signs by their own do not argue against a role of Carabin in the genetic susceptibility for SLE development in humans. Indeed, Pep619W KI mice show signs of lymphocyte hyperresponsiveness without developing pathogenic autoantibodies and signs of autoimmunity by their own (Zhang et al, 2011), although this variant has been associated to the development of autoimmune diseases, and SLE in particular, in GWAS studies (Kyogoku et al, 2004).

Despite the moderate phenotype of *Carabin*^−/−^ mice and the short window of Carabin's action on B-cell response *in vivo*, the simultaneous activation of the BCR and TLR led to autoimmunity in a subgroup of mice. Indeed, under TLR9 activation *in vivo*, anti-DNA *Carabin*^−/−^ B cells were producing autoantibodies, leading to the development of a disease similar to mesangial type II lupus glomerulonephritis (ISN/RPS classification of lupus nephritis) with a incomplete prevalence. Thus, the deficiency of Carabin could lead to a sustained production of autoantibodies by B cells, providing that a TLR9 signal is concomitantly present. These results are consistent with the role of endogenous and exogenous TLR ligands in autoantibody production as it has been shown in the AM14 Rheumatoid Factor (RF, anti-Fcγ autoantibody) transgenic murine model. AM14 B cells proliferated in response to chromatin-IgG complexes (Leadbetter et al, 2002). The same BCR/TLR dual signaling applied to anti-DNA transgenic B cells: anti-DNA 3H9 transgenic B cell proliferated in response to CpG dinucleotides (CG50) in a TLR9-specific manner (Viglianti et al, 2003). From these results, a model can be drawn in which an autoreactive B cell can be activated by autoantigenic ligands for TLR9, such as CpG, transporting the immune complexes (for RF) or the DNA (for anti-DNA BCR) to the endosome, leading to TLR9 activation. However, the frequent exposure of an individual to TLR9 ligands (endogenous or exogenous like during everyday life infectious events) without the systematic production of autoantibodies suggests the existence of a protective negative regulation of this dual signaling process. The fact that some Carabin KO and B-cell-specific Carabin KO mice immunized with CpG-DNA produce higher titers of anti-dsDNA antibodies indicates that Carabin, by regulating the Erk pathway, could be implicated in this negative regulation. Carabin could prevent the early activation of anergic or ignorant autoreactive B cells after infection-induced TLR, and notably TLR9, signaling. Further experiments will directly test this model.

Our *in vitro* experiments suggest a mechanism, which could explain how low B-cell levels of Carabin can favour overt autoimmunity after TLR9 activation: Carabin inhibits the molecular crossroad between the BCR pathway and the TLR9 pathway. A crossroad has already been demonstrated in macrophages between TLR9 and Ras (Xu et al, 2003) and whether Ras itself, or another closely related molecular partner, is directly involved merits further investigations. Our results show that, after BCR stimulation or BCR-TLR9 costimulation, the absence of Carabin in B cells accelerates Erk phosphorylation and increases *Egr1* transcription and CD44 expression, confirming the implication of Erk phosphorylation in B-cell function as suggested by Dinkel et al (Dinkel et al, 1997; 1998). Interestingly, but for possible multiple molecular reasons, Erk phosphorylation was also increased in BCR-stimulated B cells from 4-month-old (NZB × NZW)F1 mice (unpublished observations) otherwise showing a 50% decrease of *Carabin* expression.

In humans, Carabin was not to date identified as a candidate susceptibility gene during GWAS. However, the low expression of Carabin in B cells during SLE could result from an epigenetic phenomenon, which now needs to be clarified. Carabin 3′ UTR sequence does not contain any target sequences for miRNAs, which have been associated to SLE. We can also hypothesize that environmental factors (UV-light exposure, oestrogens, drugs, infections) can aggravate Carabin underexpression in SLE B cells.

Deciphering the molecular partners of Carabin in B cells as well as identifying the correlations of Carabin levels and clinical phenotypes of the lupus patients could lead to the indentification of new therapeutic targets. In addition, the cross of Carabin-deficient mice into various SLE models will be useful to better understand the mechanism by which the deficiency of Carabin could contribute to autoimmunity. Finally, it would be important to evaluate if a low expression of Carabin is also a characteristic of other autoimmune diseases. This point must be evaluated in a prospective way in new cohorts of patients.

## MATERIALS AND METHODS

### Patients

Patients with SLE fulfilling at least four diagnostic criterias according to the American College of Rheumatolog (Hochberg, 1997) were prospectively included in two consecutive cohorts provided that they were in a quiescent phase of the disease and received minimal treatment (no immunosuppressive drugs). Purified B cells from the first 17 patients (and 9 age and sex matched controls) were subjected to a pangenomic transcriptoma analysis (Affymetrix GeneChip human genome U133 plus 2.0). In the second cohort, B cells were purified from 10 patients and 10 healthy individuals, total RNAs were extracted and *Carabin* mRNA expression was determined by real time quantitative RT-PCR. This study was approved by the ethic comity of the Hôpitaux Universitaires de Strasbourg and patients gave their written informed consent.

### Cell preparation and culture

A20 cell line was from American Type Culture Collection (ATCC) and was cultured in RPMI with 10% (vol/vol) FCS (PAN). 2.10^6^ cells were stimulated with LPS alone (10 µg/ml; Sigma) or with F(ab′)_2_ anti-Mouse IgG (10 µg/ml; Jackson Immunoresearch). Splenic cells were cultured in RPMI with 10% (vol/vol) FCS (PAN). Cells were stimulated with LPS alone (10 µg/ml; Sigma), LPS (10 µg/ml) plus IL-4 (20 ng/ml Sigma), F(ab′)_2_ anti-mouse IgM (0.5, 2, 5 or 10 µg/ml; Jackson Immunoresearch), ODN2395 (1 µg/ml; Invivogen), anti-CD3 (2 µg/ml; BD Biosciences) or anti-CD3 (2 µg/ml; BD Biosciences) plus anti-CD28 antibodies (2 µg/ml BD Biosciences). Purification of splenic total, or mature B cells and splenic total T cells was done using B-cell isolation kit, CD43 (Ly-48) microbeads and Pan-T-cell isolation kit (all from Miltenyi Biotech), respectively. For the analysis of proliferation, cells were stained with CFSE (Molecular Probes) before the stimulation. Bone marrow and splenic B-cell subsets were sorted by flow cytometry with a FACS Aria cell sorter (BD Biosciences).

### Gene knockdown and A20 B cells transduction

We have used pTRIP.CMV.GFP lentiviral vector for short hairpin RNA (shRNA) delivery. The lentiviral vector was kindly provided by Dr. Pierre Charneau (Zennou et al, 2000). For the construction of pTRIP-shCarabin, a DNA fragment containing H1 promoter and Carabin shRNA sequence was generated by double digestion of pSUPER-Carabin plasmid (made by inserting a shRNA targeting murine Carabin cDNA sequence (5′-GAACTACAGGATGATTCTA-3′) into pSUPER plasmid) and was subcloned within the 3′ long terminal repeat of pTRIP.CMV.GFP vector. Lentiviral particles were produced by transient transfection of 293T cells, as previously described (Zennou et al, 2000). Viruses were then used to transduce 3.10^5^ A20 B cells in the presence of polybrene (Sigma). GFP-positive A20 B cells were sorted by flow cytometry with a FACS Aria cell sorter (BD Biosciences).

### Quantitative real-time RT-PCR analysis

mRNA was prepared with RNeasy Kit (Qiagen) and cDNA was obtained with High Capacity Reverse Transcription Kit (Applied Biosystems). Quantitative real-time PCR was performed on 10 ng cDNA using Taqman Universal Mastermix (Applied Biosystems) and Assays-on-Demand probes (*Hprt1*: Mm01318743_m1 and Hs01003267_m1, 18S: Mm03928990_g1, *Carabin*: Mm00724447_m1, *Carabin* probe 1: Mm01236554_m1, *Carabin* probe 2: Mm01236552_m1, *Carabin* probe 3: Mm01236550_g1 and Hs00736460_m1; *Egr1*: Mm00656724_m1; *TIS11b* (*Zfp36l1*): Mm01304623_g1, Applied Biosystems). Each sample was amplified in triplicate in a StepOnePlus real-time PCR machine (Applied Biosystems). mRNA levels were calculated with the StepOne v2.1 software (Applied Biosystems), using the comparative cycle threshold method, and normalized to the endogenous control *Hprt1*.

### Immunoblot analysis

Proteins were extracted by standard techniques. Primary antibodies and dilutions were as follows: anti-C-ter Carabin, 1:2000 (Abcam); anti-Actin, 1:5000 (Santa Cruz); anti-GAPDH, 1:5000 (Santa Cruz); anti-phospho-SAPK/JNK, 1:2000 (Cell Signaling); anti-SAPK/JNK, 1:2000 (Cell Signaling); anti-phospho-p44/42 (anti-phospho-Erk1/2), 1:2000 (Cell Signaling); anti-phospho-IκB-α, 1:2000 (Cell Signaling); anti-p44/42 (anti-Erk1/2), 1:2000 (Cell Signaling); anti-NFATc1, 1:1000 (Santa Cruz biotechnology). For NFAT nuclear translocation assay, cytoplasmic and nuclear extracts were prepared using the NE-PER Nuclear and Cytoplasmic Extraction Kit (Thermo Scientific).

### Mice

Generation of ES cells, *Carabin*^−/−^, and conditional *Carabin*^−/−^ mice: mice with complete Carabin deficiency were generated by introduction of loxP sites on both sides of the 4th–5th exons (Supporting Information Fig 1A and B), because the TBC domain, responsible for the Ras GAP activity, extends from the 4th to the 8th exon, and because the 5th exon contains the Arg139 (5th exon), corresponding to Arg141 in humans, and which is necessary for this activity. The targeting vector was constructed as follows. A 0.6 kb fragment-encompassing exons 4 and 5 was amplified by PCR (from 129S2/SvPas ES cells genomic DNA) subcloned in an ICS proprietary vector. This ICS vector contains a LoxP site as well as a floxed and flipped Neomycin resistance cassette. A 4.6 kb fragment (corresponding to the 5′ homology arm and 3.3 kb fragment corresponding to the 3′ homology arms were amplified by PCR and subcloned in step1 plasmid to generate the final targeting construct. The linearized construct was electroporated in 129S2/SvPas mouse embryonic stem (ES) cells. After selection, targeted clones were identified by PCR using external primers and further confirmed by Southern blot with 5′ and 3′ external probes. Two positive ES clones were injected into C57BL/6J blastocysts, and male chimaeras derived gave germline transmission. Chimeras were with Flp deletor mice for direct excision of the flipped NeoR cassette. The obtained *Carabin*^*f/+*^ mice were backcrossed on C57BL/6 background for at least 8 generations. *Carabin*^*f/+*^ mice were crossed to CMV-Cre animals to produce *Carabin*^*+/−*^ mice. Homozygous Carabin KO mice were generated by intercross of *Carabin*^*+/−*^ mice. *Carabin*^*f/+*^ mice were crossed to *Carabin*^*+/−*^ mice and then to *Mb1-Cre* (provided by M. Reth, Freiburg, Germany) and *dLck-Cre* transgenic mice (provided by N. Killeen, California, USA) to generate immature B-cell and mature T-cell Carabin conditional KO models, respectively. The presence of the *Carabin*^*−*^, -floxed (*Carabin*^*f*^) or -wt (*Carabin*^*+*^) allele was assessed in offsprings by PCR analysis using specific primers (Lf1: 5′-GCAGCACAGCAGCTACAGGTCCC-3′; Ef: 5′-GCGCCACCATTGCCCAGCTCTA-3′; Er1: 5′-CCCTCTGCAGACCTCATCCGCC-3′). The validation of exons 4–5 deletion strategy was performed on *Carabin*^−/−^ mice. Carabin mRNA expression was analysed by quantitative real-time RT-PCR in splenocytes from *Carabin*^−/−^ mice using different probes, showing an complete absence of production of any transcript containing exons 4–5, but the production of transcripts containing exons 2–3 or exons 6–7 (Supporting Information Fig 1C). This suggests the expression of a shorter transcript in *Carabin*^−/−^ mice compared with *Carabin*^*+/+*^ mice, which could be the result of an out-of-frame splicing of exon 4–6, generating a premature stop codon immediately downstream exon 6. In addition, Western-Blot analysis failed to detect any Carabin expression in *Carabin*^−/−^ splenocytes and thymocytes (Supporting Information Fig 1D). *Carabin* flox deletion was almost complete in the corresponding B or T cells, as shown by PCR and Western-Blot analysis on purified splenic B or T lymphocytes, respectively, without any deletion in thymocytes (Supporting Information Fig 1E and F). (NZB × NZW)F1 mice were obtained from Harlan. All animal experiments were performed with approval by the Direction départementale des services vétérinaires (Strasbourg, France) and protocols were approved by the Comité d'éthique en matière d'Experimentation Animale de Strasbourg (CREMEAS, approval number AL/02/15/09/11). All control mice used in the experiments were littermate controls.

**The paper explained**

PROBLEM:

SLE is a systemic, frequent and severe autoimmune disease characterized by the production of various pathogenic autoantibodies, which participate in multi-organ damage. The aetiology of SLE is both genetic and environmental. B-lymphocytes play a central role in the disease and carry intrinsic genetic defects, which would make an individual more susceptible to the development of autoimmunity. However, only a few genes have been validated in humans and it is important to identify new gene defects that could be responsible for autoimmunity.

RESULTS:

We investigated gene expression abnormalities in B cells from SLE patients and from lupic mice and have identified a similar decrease in the expression of *Carabin* gene. Here, we have studied the role of Carabin in B cell and in the development of autoimmunity using Carabin-deficient cells and mice. We have first shown that Carabin is a new negative regulator of one the most important BCR-dependent signalling pathways, Erk. Its deficiency in B cells accelerates the production of specific antibodies after immunization with classical antigens, and makes the mice more susceptible to the production of autoantibodies and the development of signs of renal damages after immunization with CpG-DNA, mimicking a viral infection by activation of TLR9 receptor (belonging to the TLR family of receptors recognizing pathogen components). We further showed that Carabin deficiency leads to a higher Erk activation after TLR9 and BCR costimulation in B cells.

IMPACT:

This study identified the deficiency of Carabin expression as a novel gene expression abnormality in SLE B cells. Carabin deficiency could make an individual more susceptible to the development of autoimmunity. These results are important for a better comprehension of biological actors implicated in SLE development. This could give new opportunities into the development of new diagnostic and therapeutic tools.

### Flow cytometry analysis

Cell phenotype was performed on thymic, splenic, and bone marrow lymphoid populations by four-colour fluorescence analysis according to standard protocols. The following antibodies and reagents were used: FITC, PE, or APC anti–mouse B220, CD3, CD4, CD8, CD19, CD21, CD23, CD44, CD69, CD86, I-A/I-E, CD25 and IgM (all from BD Biosciences), biotin-OVA (5 µg/ml), Cy5-streptavidin (Jackson Immunoresearch). Propidium iodide was used for live-dead discrimination. Intracellular staining was performed as previously described (Krutzik & Nolan, 2003). Antibody used for intracellular staining was: anti-Erk1/2 (BD Biosciences). Cells were analysed using a FACSCalibur. We then analysed data with FlowJo software (Treestar).

### Calcium mobilization assay

Splenic purified (CD43-negative) mature B cells were loaded with Indo-1 (4.5 µM; Molecular Probes) at 37°C for 45 min in IMDM, 1% FCS. After washing, cells were prewarmed at 37°C before analysis. Intracellular calcium (Ca^2+^) levels (ratio of Indo-1 violet/blue) of purified B cells were then analysed by flow cytometry using FACSAria (Becton Dickinson). Data were collected for 120 s to establish baseline violet/blue ratios; cells were then stimulated by the addition of 5 µg/ml anti-mouse IgM (Jackson Immunoresearch) or 1 µM ionomycin (Cell Signaling) and data were collected for five additional minutes.

### Antibody detection by ELISA

Total IgG, IgG1, IgG2b, IgG3 or IgM levels were measured in serum from 8- to 12-week-old mice, and in supernatants after 3 days of stimulation, as previously described (Soulas-Sprauel et al, 2007). The wells were developed with Fast OPD substrate (Sigma–Aldrich). Absorbance was measured at 490 nm. Levels of Ig were determined by comparison with a standard curve using purified IgG, IgG1, IgG2b, IgG3, IgMκ (Sigma–Aldrich; Southern Biotechnology Associates). The serum reactivity was tested with thyroglobulin, dsDNA, actin as previously described (Koenig-Marrony et al, 2001). To measure anti-OVA or anti-NP specific antibodies, 96-wells ELISA plates were coated with OVA (50 µg/ml, Sigma) and NP-bovine serum albumin (BSA) (5 µg/ml, Biosearch Technologies) respectively, in sodium carbonate buffer (pH 9.6). The wells were blocked with 1% BSA for 30 min at 37°C and incubated with diluted sera for 2 h at 37°C. Horseradish peroxidase-conjugated isotype-specific antibodies (all from Interchim) were used as revealing antibodies. For anti-OVA competitive inhibition ELISA assay, the previous ELISA was modified by prior incubation of sera with increasing concentrations of soluble OVA, according to Friguet's method (Friguet et al, 1985).

### Immunization and infection

Six- to eight-week-old mice were injected intraperitoneally (i.p.) and bled on days indicated in the figures. Mice were injected with 100 µg Ovalbumin (Sigma) in complete Freund's adjuvant (Sigma) or with 100 µg NP-LPS (Biosearch Technologies) in PBS. For CpG-DNA treatment, 8-week-old mice were injected i.p., every other day for 2 weeks, with 40 µg CpG ODN 2395 (tcgtcgttttcggcgcgcgccg) with phosphorothioate bases (Invitrogen). Serum was collected before treatment, and every weeks after the first injection. *Borrelia burgdorferi* (Bb) infection and quantification of anti-Bb IgG response were done as described (Soulas, 2005).

### Histology and Immunohistochemistry

Kidneys were fixed in 4% formaldehyde and embedded in paraffin. Sections and Hematoxylin/Eosin (HE) and Trichrome (T) stain were performed. For immunohistochemistry, kidneys were embedded in Tissue-Tek OCT compound and snap frozen in methyl-butane with liquid nitrogen. Tissue sections were then stained with IgG-FITC (Jackson Immunoresearch), and IgM-biotin (Beckman Coulter) followed by streptavidin-Alexa546 (Molecular Probes). Samples were analysed by an anatomopathologist.

### Statistical analysis

Statistical significance was calculated with a two-tailed Mann & Whitney test or a Fisher exact's test, using Prism software (GraphPad).

## Supplementary material

Detailed facts of importance to specialist readers are published as ”Supporting Information”. Such documents are peer-reviewed, but not copy-edited or typeset. They are made available as submitted by the authors.
