# Supplementary material for: Carabin deficiency in B cells increases BCR-TLR9 costimulation-induced autoimmunity
Source: EMBO Mol Med. 2012 Oct 29;4(12):1261–75. doi: 10.1002/emmm.201201595 (PMC3531602; doi:10.1002/emmm.201201595)
Supplement: Supplementary file 1 [file emmm0004-1261-SD1.pdf]

EMBO
Molecular Medicine

Manuscript EMM-2012-01595

Carabin deficiency in B cells increases BCR-TLR9 costimulation-induced autoimmunity

Jean-Nicolas Schickel, Jean-Louis Pasquali, Anne Soley, Anne-Marie Knapp, Marion Decossas, AurÈlie Kern, Jean-Daniel Fauny, Luc Marcellin, Anne-Sophie Korganow, Thierry Martin and Pauline Soulas-Sprauel.

Corresponding author: Pauline Soulas-Sprauel, CNRS UPR9021

Review timeline:

Submission date:	24 May 2012
Editorial Decision:	26 June 2012
Revision received:	31 July 2012
Editorial Decision:	23 August 2012
Revision received:	19 September 2012
Accepted:	21 September 2012

Transaction Report:

(Note: With the exception of the correction of typographical or spelling errors that could be a source of ambiguity, letters and reports are not edited. The original formatting of letters and referee reports may not be reflected in this compilation.)

1st Editorial Decision 26 June 2012

Thank you for the submission of your manuscript "Carabin deficiency in B cells increases BCR-TLR9 costimulation-induced autoimmunity" to EMBO Molecular Medicine. We have now heard back from the referees whom we asked to evaluate your manuscript. You will see that they find the topic of your manuscript potentially interesting. However, they also raise significant concerns on the study, which should be addressed in a major revision of the manuscript.

In particular, both Reviewers highlight that further mechanistic insight should be provided. In this respect, Reviewer #1 notes that the evidence for the negative regulatory activity of carabin on BCR stimulation should be strengthened while Reviewer #2 inquires about the regulation of carabin expression. Importantly, Reviewer #1 highlights similar concerns in point 1, which should be convincingly addressed.

On a more editorial note, EMBO Molecular Medicine does not permit citation of "Data not shown". All data referred to in the paper should be displayed in the main or supporting figures. "Unpublished observations" may be referred to in exceptional cases, where these are data peripheral to the major message of the paper and are intended to form part of a future or separate study. In addition, the description of all reported data that includes statistical testing must state the name of the statistical test used to generate error bars and P values, the number (n) of independent experiments underlying each data point (not replicate measures of one sample), and the actual P value for each test (not merely 'significant' or 'P < 0.05'). Please see our Guide to Authors for further information.

Given the balance of these evaluations, we feel that we can consider a revision of your manuscript if

you can convincingly address the issues that have been raised within the space and time constraints outlined below.

Revised manuscripts should be submitted within three months of a request for revision. They will otherwise be treated as new submissions, unless arranged differently with the editor.

I look forward to seeing a revised form of your manuscript as soon as possible.

Yours sincerely,

Editor
EMBO Molecular Medicine

***** Reviewer's comments *****

Referee #1:

Schickel et al observed low carabin levels in NZB/W F1 mice and in clinically inactive SLE patients. They created KO and conditional KO of this gene in mice and observed no change in T or B cell numbers and no spontaneous activation. However, increased Erk phosphorylation in both B and T cells was observed and a mild lupus-like phenotype induced by CpG administration, which required carabin deficiency in B, but not T cells. They conclude that carabin plays an important role in inhibition of B cell activation in the context of TLR stimulation. This work is highly original and quite thorough.

1. There are some theoretical objections to the authors conclusions. In Fig. 2, they observe that carabin levels decline following BCR stimulation. This raises the important question as to whether the low carabin in SLE patients and mice is a consequence of activation. SLE patients with low SLEDAI may still have high titers of autoantibodies as well as many signs of subclinical immune activation. Similarly, NZB/W mice at 4 months of age often already have autoantibodies. One would need to see carabin levels (or even better some genetic explanation) at 2 months of age, to be more convinced that low levels came first.
Since the authors show that immature B cells (T1, T2) also show lower carabin levels compared to mature cells, a second possibility is that the low carabin levels reflect higher numbers of circulating immature B cells in lupus. More careful phenotypic analysis of the B cells in SLE patients studied would help resolve this issue.

2. The major hypothesis here is that carabin is a negative regulator of BCR stimulation but the only evidence provided is increased Erk phosphorylation. How do the authors exclude more upstream signal like calcium flux? Why not also overexpress carabin and test whether it suppresses activation / proliferation / maturation?

3. What is the result of in vitro B cell stimulation by TLR9 or TLR9 plus anti-BCR?

4. Fig. 6. Kidney sections should also be stained for C3. Also, if the pathology is thought to be mesangial, the kidneys should be compared after PAS staining.

5. Why are so few mice examined for the lupus phenotype? Numbers should at least be doubled to be convincing.

Other comments
All figures should state the numbers of mice studied. Where possible e.g. Figs. 4 and 7, results should be expressed as the average of 3 experiments rather than representative data -otherwise no meaningful statistical analysis can be performed.

p.10: what does "normal overexpression" mean in the sentence "1) Carabin -/- B cells proliferated normally in response to BCR-dependent (anti-IgM) or BCRindependent
(LPS) stimulation (Fig 4C) and displayed a normal overexpression of activation
markers (CD86, CD69, MHCII) compared to control Carabin +/+ mice (Fig 4D)"?

© EMBO

Fig. 8 is only moderately helpful and could be put in Suppl Figs. The right panel should show absence by a cross over the carabin. The figure doesn't actually resemble the data but only speculation as, in the experiments performed, DNA enters the cell through a non BCR route.

It would be helpful to know (or even speculate) how BCR and TLR signal transductions pathways intersect.

Referee #2:

The authors studied the potential role of Carabin, a negative regulator of T and B cell activation, in the pathogenesis of SLE. Starting with the observation that Carabin expression is low in B cells of (NZBxNZW) F1 mice long before disease onset, and in B cells of lupus patients during the inactive phases of disease the authors analysed B cell responses of a Carabin KD A20 cell line and Carabin KO mice for anti-DNA antibody production und development of lupus nephritis. They come up with a very attractive model for a polygenic , multi-hit pathophysiology of SLE involving both activation of autoreactive B cells via their BRC and TLR9 co-stimulation thereby mimicking a viral co-infection. Carabin KO or KD does not cause per se an increased activation phenotype of B cells, although Carabin deficiency in B cells speeds up early B cell responses and makes the mice more susceptible to anti-dsDNA production and renal lupus flare upon stimulation with a TLR9 agonist, CpG-DNA. By elegant in vitro studies of the NFkB and ERK phosphorylation pathways in B cells the authors convincingly identify Carabin as a natural inhibitor of a potentially dangerous cross-talk between BCR and TLR9 pathways in autoreactive B cells.

The authors confirm and extend work from Pan et al (2007) that Carabin -/- T cells show an increased T cell activation pattern in vitro with enhanced proliferation, increased basal expression of IL2 and activation markers (CD25, CD44) and a further increase of CD69 and CD25 expression upon stimulation with anti-CD3 + antiCD28; ERK phosphorylation was similarly enhanced as in activated B cells.
Carabin -/- mice exhibit an accelerated antibody response in vivo to T-dependent and T-independent antigens indicating an accelerated class switch mechanism and a facilitated antigen-specific affinity maturation. Most fascinating is the observation that Carabin-/- mice develop signs of autoimmunity upon immunisation with CpG with appearance of anti-dsDNA antibodies and immune complex deposition in the kidneys. Interestingly (NZBxNZW)F1 lupus-prone mice exhibit at month 4 (before disease onset) a 50% reduced Carabin expression (confirmed by RT-PCR) compared to controls and like the A20 Carabin KD B cells they show a strongly enhanced ERK phosphorylation.

Major comments:
The negative regulator effects of Carabin at the intersection between BCR and TLR9 signalling pathways have been well characterized, however, the manuscript contains only little information on the regulation of this rather crucial natural inhibitor.
1. It would be most interesting to get some ideas on what might brings Carabin expression down a priori? Could lupogenic events play a role such as UV-light exposure?, estrogens? Anti-convulsive drugs (e.g. anti-epileptica)? Tissue damage due to polytrauma or infections?
2. Are there epigenetic regulations operative in reducing Carabin expression in SLE?
3. Is low Carabin expression restricted to SLE or has it been found also in other systemic autoimmune diseases such as Sjˆgren's syndrome, scleroderma, RA a.o.?

Minor comments:
Abbreviations are widely used in the manuscript. Their definitions are not always clear. Similarly, style of language can be improved in some passages of the manuscript by having it read by a native English speaker.

Overall
Very innovative and highly attractive pathophysiological model of murine and human SLE. Worth a publication in EMBO Molecular Medicine after revision.

1st Revision - Authors' Response 31 July 2012

Referee 1:

1. There are some theoretical objections to the authors conclusions. In Fig. 2, they observe that carabin levels decline following BCR stimulation. This raises the important question as to whether the low carabin in SLE patients and mice is a consequence of activation. SLE patients with low SLEDAI may still have high titers of autoantibodies as well as many signs of subclinical immune activation. Similarly, NZB/W mice at 4 months of age often already have autoantibodies. One would need to see carabin levels (or even better some genetic explanation) at 2 months of age, to be more convinced that low levels came first.

We feel confident with the fact that the low Carabin expression in SLE B cells from these inactive patients does not reflect B cell activation. Indeed, our laboratory has done a concomitant extensive immunophenotyping of B cells in the same patients' samples used for the transcriptoma analysis mentioned in our article (Korganow A-S et al, J Autoimmun, 2010, 34, 426-434): the mean fluorescence intensity (MFI) was not different for CD86 expression in patients (CD86 MFI, patients group: 10.45+/-5.31) versus healthy donors (CD86 MFI: 7.9+/-2.64). This has been added in the results section, page 7, lines 15-16.

This point being important, we followed the referee's recommendation and analysed by real-time quantitative RT-PCR the expression of Carabin in splenic purified B cells from 2 month-old BW mice, long time before the appearance of B cell hyperactivation, autoantibodies and disease. We confirmed a 43% reduction of Carabin expression in BW mice compared to Balb/c mice ($p<0.05$). This has been added in the results section, page 7, lines 6-8, and in Figure 1A.

However, the precise mechanism, which is responsible for Carabin a priori down regulation in SLE, remains obscure, is an entire new field, and will be the subject of intense efforts.

Since the authors show that immature B cells (T1, T2) also show lower carabin levels compared to mature cells, a second possibility is that the low carabin levels reflect higher numbers of circulating immature B cells in lupus. More careful phenotypic analysis of the B cells in SLE patients studied would help resolve this issue.

As mentioned above, an extensive immunophenotyping analysis of the B cells in the studied SLE patients has been done: among the CD19+ cells, CD27- CD23- cells, that include more immature B cells (and notably transitional B cells) represent 33+/-16.5 % of CD19+ cells in patients, and 27.2+/-10.9% of CD19+ cells in healthy controls. This difference is not statistically significant ($p=0.4$, Mann&Whitney test), and cannot account for the low Carabin expression that we observe in the SLE patients. In addition, in an on-going study in which we performed a more detailed B cell phenotyping during SLE, peripheral blood transitional (CD19+, $CD38^{hi}$, IgM^{hi}) B cells represent less than 5% of total B cells. Such a small population cannot explain the low Carabin level in SLE quiescent patients.

2. The major hypothesis here is that carabin is a negative regulator of BCR stimulation but the only evidence provided is increased Erk phosphorylation. How do the authors exclude more upstream signal like calcium flux?

Pan et al (Nature, 2007, 445, 433-436) have shown that Carabin has a dual inhibitory activity on Calcineurin and Ras pathways In T cells. Our hypothesis is that Carabin plays the same role in B cells. In the previous version of our article, we confirmed that Carabin is also an inhibitor of Ras/Erk pathway in B cells (Figures 3 and 4). As asked by the referee 1, and in order to better confirm that Carabin is a negative regulator of B cells, we analysed the translocation of NFAT from the cytoplasm to the nucleus in *Carabin -/-* B cells, after anti-IgM stimulation. Our results confirm the data obtained by Pan et al, and reflect an inhibitory activity of Carabin on Calcineurin pathway in B cells. This has been added in the results section, page 10, lines 24-25, and page 11, lines 1-3, and in Figure 4G.

However, we also followed the referee's recommendation to exclude that Carabin acts on more upstream signals, like calcium influx. As shown in Supporting Information Fig 7A and B, although the basal cytoplasmic calcium level seems to be slightly increased in Carabin KO B cells, the calcium flux is normal in *Carabin -/-* B cells, compared to *Carabin +/+* B cells, after anti-IgM or ionomycin stimulation. This has been added in the results section, page 11, lines 3-4, and in Supporting Information Fig 7.

Why not also overexpress carabin and test whether it suppresses activation / proliferation / maturation?

This point is interesting and has been studied by Pan et al (Nature, 2007, 445, 433-436) in T cells: they have shown that Erk phosphorylation is accelerated in a stable Jurkat T-cell line expressing the N-terminal Ras GAP domain of Carabin. However, in a translational effort from humans to mice, the natural objective of our study was mostly designed to reproduce the pathologic condition in SLE quiescent patients, and to analyse the consequences of Carabin under expression on B cell phenotype. However, we agree that in future experiments, it could be interesting to investigate the possible therapeutic effects of manipulating the Carabin levels in lymphocytes.

3. What is the result of in vitro B cell stimulation by TLR9 or TLR9 plus anti-BCR?

Our results show that CpG-DNA induced an increased and also sustained Erk phosphorylation in BCR-stimulated B cells in *Carabin -/-* mice (Fig 7C, and D). In addition, the expression of target gene Egr1 was also increased by the costimulation (Fig 7E). A direct link of Egr1 overexpression and the expression of B cell activation markers is today unknown for CD86 and MHC II. For CD69, it has been described that the promoter region of human CD69 gene contains putative binding sequences for Egr1 (Lopez-Cabrera M., J Biol Chem, 1995, 270, 37, 21545-21551). However, CD69 is very slightly expressed in activated B cells, and we did not detect any difference in CD69 expression in anti-IgM+CpG DNA costimulated B cells in *Carabin -/-* mice, compared to *Carabin +/+* mice. For CD44, interesting results have been published by Maltzman J.S. et al (Mol Cell Biol, 1996, 16, 5, 2283-2294), showing that Egr1 regulates stimulus-dependent CD44 transcription in B lymphocytes. Therefore we analysed CD44 expression on splenic *Carabin -/-* and +/+ purified B cells, after 6h and 12h stimulation with anti-IgM and CpG DNA: our results show that the costimulation induces an increase of CD44 expression that is superior in *Carabin -/-* B cells, compared to *Carabin +/+* B cells. This has been added in the results section, page 14, lines 23-25, and page 15, lines 1-2, and in Supporting Information Fig 12.

4. Fig. 6. Kidney sections should also be stained for C3. Also, if the pathology is thought to be mesangial, the kidneys should be compared after PAS staining.

We have shown that in some CpG DNA treated *Carabin -/-* and B cell specific *Carabin -/-* mice with high score of renal IgG deposits, classical histological analysis was very similar to mesangial type II glomerulonephritis with an increase of mesangium cellularity (ISN/RPS classification) (Fig 6).

The referee's recommendation was to test for C3 deposits. This recommendation clearly improved our data since we observe a correlation between the scores of IgG and C3 deposits. This has been added in the results section, page 13, lines 21-22, and in Fig 6A.

According to the ISN/RPS classification of lupus nephritis (Weening et al, J Am Soc Nephrol, 2004, 15, 204-250), "Class II lupus nephritis is defined as mesangial proliferative lupus nephritis characterized by any degree of mesangial hyper cellularity in association with mesangial immune deposits". We think that our results reach these prerequisites, because of the observed mesangial cellularity and both anti-IgG and anti-C3 stainings.

5. Why are so few mice examined for the lupus phenotype? Numbers should at least be doubled to be convincing.

We understand the referee's comment. However, we have added statistics in the Table 1, showing the production of anti-dsDNA and deposition of IgG in renal glomeruli in CpG-DNA treated *Carabin -/-* and B cell specific *Carabin -/-* mice: despite the small numbers of experimental animals, the proportions of mice showing the highest score of renal IgG deposits are statistically different between control mice and B cell specific Carabin deficient mice. In addition, we are confident with the data because there is a correlation, animal per animal, between anti-dsDNA titer, renal IgG deposits, and now renal C3 deposits.

Altogether, we think that increasing the number of analysed mice is not mandatory to validate our conclusion.

The referee 1 proposes that we inject a higher number of mice with CpG-DNA (Table 1). This experiment requires 2-month old Carabin -/-, +/+ and B cell specific Carabin -/- mice, and mice are analysed at day 49 after the first injection. Then, the immunohistological and immunofluorescent analysis and quantification of autoantibodies by ELISA have to be made. Considering the time frame of 3 months for the submission of a revised version of our manuscript, et and the difficulty to obtain Carabin -/- and especially B cell specific Carabin -/- mice, otherwise being exactly 2-month old at the beginning of the experiment, we can't make this additional experiment.

We also apologize for a mistake which was present in the initial Table 1, concerning the anti-dsDNA IgG titers in *Carabin -/-* mice: the percentages did not correspond to the total number of analysed mice, but this did not change the message. The correction was made in the new Table 1.

Other comments:
All figures should state the numbers of mice studied.
When it was missing, we have added in the figures' legend the number of analysed mice.

Where possible e.g. Figs. 4 and 7, results should be expressed as the average of 3 experiments rather than representative data -otherwise no meaningful statistical analysis can be performed.
For Figure 4, we thought that the representation of the results as a FACS histogram was visually more appealing. However, we understand the referee's recommendation, we added a new Supporting Information Fig 3, showing the results as bars, and the corresponding statistical analysis. A statistical analysis has also been added in Figure 7D.

p.10: what does "normal overexpression" mean in the sentence "1) Carabin -/- B cells proliferated normally in response to BCR-dependent (anti-IgM) or BCR independent (LPS) stimulation (Fig 4C) and displayed a normal overexpression of activation markers (CD86, CD69, MHCII) compared to control Carabin +/+ mice (Fig 4D)"?
This sentence has been rephrased: …"the increase of expression of activation markers (CD86, CD69, MHCII) on B cells (Fig 4D, and Supporting Information Fig 3C) in response to BCR-dependent (anti-IgM) or BCR-independent (LPS) stimulation is not different in *Carabin -/-* and in control *Carabin +/+* mice".

Fig. 8 is only moderately helpful and could be put in Suppl Figs. The right panel should show absence by a cross over the carabin. The figure doesn't actually resemble the data but only speculation as, in the experiments performed, DNA enters the cell through a non BCR route.
We do agree with the referee on this point. The figure has been modified and has been put in Supporting Information, as Supporting Information Fig 13.

It would be helpful to know (or even speculate) how BCR and TLR signal transductions pathways intersect.
We think that this remark is a little bit contradictory with the referee 2 comment: "The negative regulator effects of Carabin at the intersection between BCR and TLR9 signaling pathways have been well characterized". As mentioned in the discussion part, only one article described a crossroad between Ras and TLR9 pathway, and it was in macrophages. This point is detailed in page 17. Furthermore, we think that we have detailed the important literature on BCR and TLR9 crosstalks in discussion part (page 18).

Referee 2:

1. It would be most interesting to get some ideas on what might brings Carabin expression down a priori? Could lupogenic events play a role such as UV-light exposure?, estrogens? Anti-convulsive drugs (e.g. anti-epileptica)? Tissue damage due to polytrauma or infections?
All these points are very interesting, but are today impossible to evaluate in the patients a posteriori, and cannot be easily tested in our animal models, even though the CpG-DNA stimulation was chosen to mimic a viral infection. These questions could be the subject of en entire new research program. We have added a sentence at the end of the discussion part (page 19, lines 9-11), opening new questions on the possible causes of Carabin low expression in SLE patients.

2. Are there epigenetic regulations operative in reducing Carabin expression in SLE?
In order to answer to this interesting question, we have searched for the existence of miRNA target sequences in Carabin 3' UTR sequence, using 3 different databases (Target Scan, Miranda, Pictar), and found potential target sequences for 7 miRNAs: miR-93, miR-20b, miR-106a, miR-20a, miR-17, miR-106b, miR519d.
Then we have searched for some associations between SLE and these miRNAs in the literature: none has ever been clearly associated with SLE (Ceribelli, A. et al, Arth Res & Ther, 2011, 13, 4, 229; Amarilyo G. et al, Clin Immunol, 2012, 144, 26-31; Pauley K.M. et al, J Autoimm, 2009,

32, 189-194; Brooks W.H. et al, J Autoimmun, 2010, 34, J207-J219). MiR-17 and miR-20a belong to the miR17-92 cluster (containing 6 miRNAs), which is up regulated in splenic T cells, but not splenic B cells, from MRL-lpr mice (Dai R. et al, Plos One, 2010, 5, 12 1-8).
Concerning this point, a new sentence has been added at the end of the discussion part (page 19, lines 8-9).
In addition, we have no idea about the regulation of Carabin expression by DNA methylation. On the other hand, increased Erk phosphorylation due to low Carabin expression could have some impact on DNA methylation.

3. Is low Carabin expression restricted to SLE or has it been found also in other systemic autoimmune diseases such as Sjögren's syndrome, scleroderma, RA a.o.?
According to the literature, Carabin has not been found under expressed in other autoimmune diseases. However, data of transcriptoma analysis of B cells in other autoimmune diseases (Sjögren, Rheumatoid Arthritis, Sleroderma) are missing. It would be very important to know if Carabin low expression plays a more general role in the physiopathology of autoimmune diseases. This point must be evaluated in cohorts of patients in a prospective way, in Rheumatoid Arthritis, Scleroderma, and Sjögren's disease (and to compare quiescent and active phases for example in Rheumatoid Arthritis).
This point has been added in the discussion part in page 19, lines 16-18.

Minor comments:

Abbreviations are widely used in the manuscript. Their definitions are not always clear. Similarly, style of language can be improved in some passages of the manuscript by having it read by a native English speaker.
Abbreviations have been simplified and clarified.
Finally, the manuscript has now been read by a native English speaker.

We hope that you will find our revised version of the manuscript suitable for publication in the *EMBO Molecular Medicine.*

2nd Editorial Decision 23 August 2012

Thank you for the submission of your revised manuscript "Carabin deficiency in B cells increases BCR-TLR9 costimulation-induced autoimmunity" to EMBO Molecular Medicine. We have now received the enclosed reports from the referees whom we asked to re-assess it.

As you will see, both Reviewers acknowledge that the manuscript was significantly improved during revision. However, while reviewer #2 indicates that that the manuscript is suitable for publication after minor points have been addressed, reviewer #1 raises technical issues that should be convincingly addressed. Since we do acknowledge the potential interest of your findings, we would therefore be open to allow a second revision of the manuscript that would address the outstanding issues.

On a more editorial note, immunoblots should be surrounded by a black line to indicate the borders of the blot, if the background is faint. Please include these in the respective figures.

I look forward to reading a new revised version of your manuscript as soon as possible.

Yours sincerely,

Editor
EMBO Molecular Medicine

***** Reviewer's comments *****

Referee #1:

Failure to examine the effect of increased expression of carabin is disappointing. On the other hand, the addition of data on young BW lupus mice, NFAT and C3 strengthens the interpretations in the manuscript. The authors do need to apply the same rigor to new data as to the original data and therefore need to attend to the following:

1. For C3, the authors use the term "correlation" inappropriately. They only show the results on two mice - the other two could be completely discordant. Since they are dealing with such a small number of mice (as mentioned previously) they need to add the aggregate data on the 4 mice to Table 1.

2. The new NFAT data has not been thoroughly analyzed. Again, a summary of several experiments with the intensity of bands normalized to control is necessary to be believable to the readers.

3. The authors assert that CD44 is upregulated more following costimulation in carabin deficient compared to wildtype mice (supporting Information Fig 12A, B). However, no statistical validation of this comment is attempted. This should be corrected.

Referee #2:

The authors have very thoroughly revised their manuscript according to the reviewers' suggestions. Thereby they have convincingly outlined a negative regulator role of Carabin in BCR and TLR9 induced B cell stimulation. The potential role of low Carabin expression levels in pre-lupus states of NZB/NZW mice and in clinically inactive SLE patients is most intriguing and promising with respect to new physiopathology horizons in SLE research.
The revised manuscript clearly merits to be published in EMBO Molecular Medicine. I have only three minor suggestions to further improve the manuscripts.
1. The authors underlined in an additional experiment the role of the sustained ERK expression in B cells from Carabin-/- mice by studying the up-regulating effect of ERK target gene Egr-1 on downstream B cell activation marker CD44. In this context their arguments might be further supported by citing the work of Dinkel al (1997, 1998) who showed that Egr-1 over-expression in B cells not only up-regulates CD44 but also down-regulates CD95 /Fas and CD23 and advances B cell differentiation at the pre-B1 and immature stage. This information corroborates the facilitating effect of an activated ERK pathway on regular and autoimmune B cell responses.
2. Typo: Page 12 , 3rd line from bottom: "... nor did they develop ..."
3. Style: Page 18, line 9-10 from top: "From these results a model can be drawn ..."

2nd Revision - Authors' Response 19 September 2012

Referee 1:

Point 1.
In order to clarify our results, we have added in Table I the data, animal per animal. We can globally observe a good correlation between serum anti-dsDNA titers, renal IgG deposits, and renal C3 deposits.

Point 2.
In order to follow the referee’s recommendation concerning the NFAT pathway, we repeated the experiment and Figure 4H is now showing the results of 3 experiments as bars, with the corresponding statistical analysis.

Point 3.

Concerning the data on CD44 up regulation, we repeated again the *in vitro* experiment (n=4) and added a statistical analysis, in Supporting Information Figure 12.

Referee 2:

We have followed the suggestions of referee 2 (point 1, text in red in page 14 last line, and in page 19, the first four lines) and corrected the mistakes (points 2 and 3).

We hope that you will find our revised version of the manuscript suitable for publication in *EMBO Molecular Medicine.*
