# Supplementary material for: Carabin deficiency in B cells increases BCR-TLR9 costimulation-induced autoimmunity
Source: EMBO Mol Med. 2012 Oct 29;4(12):1261–75. doi: 10.1002/emmm.201201595 (PMC3531602; doi:10.1002/emmm.201201595)
Supplement: Supplementary file 2 [file emmm0004-1261-SD2.pdf]

Supporting Information Table of Contents

Supporting Information Figure 1	Page 2
Supporting Information Figure 2	Page 4
Supporting Information Figure 3	Page 5
Supporting Information Figure 4	Page 6
Supporting Information Figure 5	Page 7
Supporting Information Figure 6	Page 8
Supporting Information Figure 7	Page 9
Supporting Information Figure 8	Page 10
Supporting Information Figure 9	Page 11
Supporting Information Figure 10	Page 12
Supporting Information Figure 11	Page 13
Supporting Information Figure 12	Page 14
Supporting Information Figure 13	Page 15
Supporting Information Table 1	Page 16

Supporting Information Figure 1

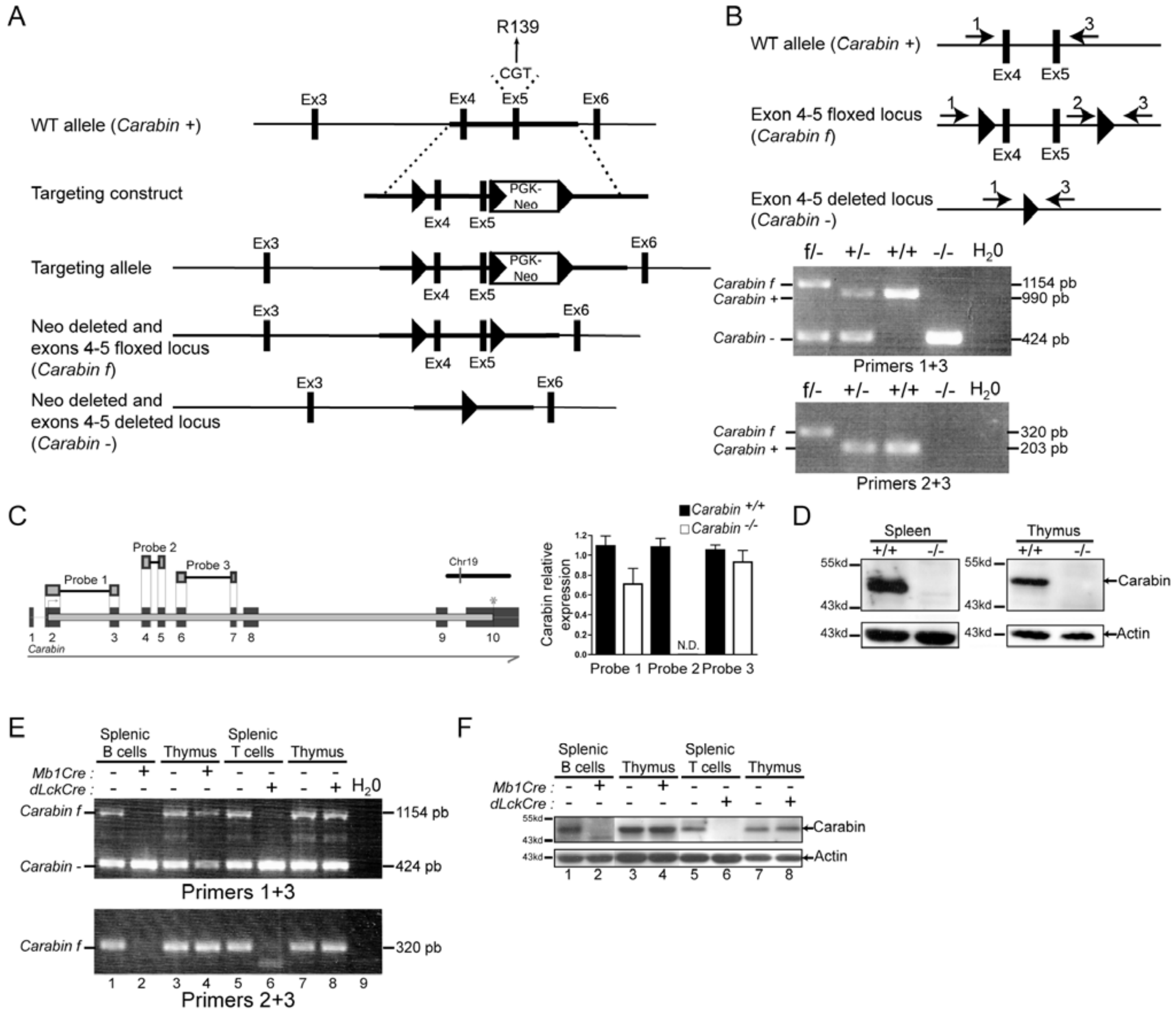

Supporting Information Figure 1. *Carabin* gene targeting. (**A**) Schematic representation of the targeted region of murine *Carabin* locus (*Carabin* +), targeting construct, targeting allele, Neo-deleted and exons 4-5 floxed (*Carabin f*) allele, and Neo-deleted and exons 4-5 deleted (*Carabin* -) allele. Filled boxes represent exons; filled triangles, LoxP sites. Note that the NeoR cassette is flipped and floxed (FRT sites are not represented) (**B**) Screening of *Carabin f/-*, *Carabin +/-*, *Carabin +/+* and *Carabin -/-* mouse line progenies by PCR on tail DNA, using primers 1 (Lf1), 2 (Ef) and 3 (Er1); see “Generation of ES cells, *Carabin -/-*, and conditional *Carabin -/-* mice” in the Methods section. Filled box represents exons; filled triangles, LoxP sites. Primers 1 and 3 reveal a 1.15 kb floxed (*Carabin f*), a 0.99 kb WT

(*Carabin* +) and a 0.42 kb deleted (*Carabin* -) alleles, respectively. Primers 2 and 3 reveal a 0.20 kb WT and 0.32 kb floxed alleles, respectively. (**C**) Quantitative real-time RT-PCR analysis of *Carabin* mRNA expression in splenocytes from *Carabin* +/+ or *Carabin* -/- mice, using three different probes matching exons 2-3 (Probe 1), exons 4-5 (Probe 2), exons 6-7 (Probe 3). Each sample was normalized to the endogenous control Hprt1. (**D**) Western blot analysis of splenic and thymic cells from *Carabin* +/+ or *Carabin* -/- mice. Actin was used as loading control. (**E**) PCR analysis of *Carabin* deletion. *Carabin f* and *Carabin* - alleles were amplified from genomic DNA of purified splenic total B or T cells (depending on the conditional KO model), and thymocytes from *Carabin f/-* mice expressing Cre (+) or not (-), using a combination of primers 1, 2, and 3 (depicted in Supplementary Figure 1b). (**F**) Immunoblot analysis of Carabin expression in purified splenic total B or T cells, and thymocytes from *Carabin f/-* mice expressing Cre (+) or not (-), using an anti-Carabin antibody. Actin was used as loading control. (**C**; errors bars, standard deviation).

Supporting Information Figure 2

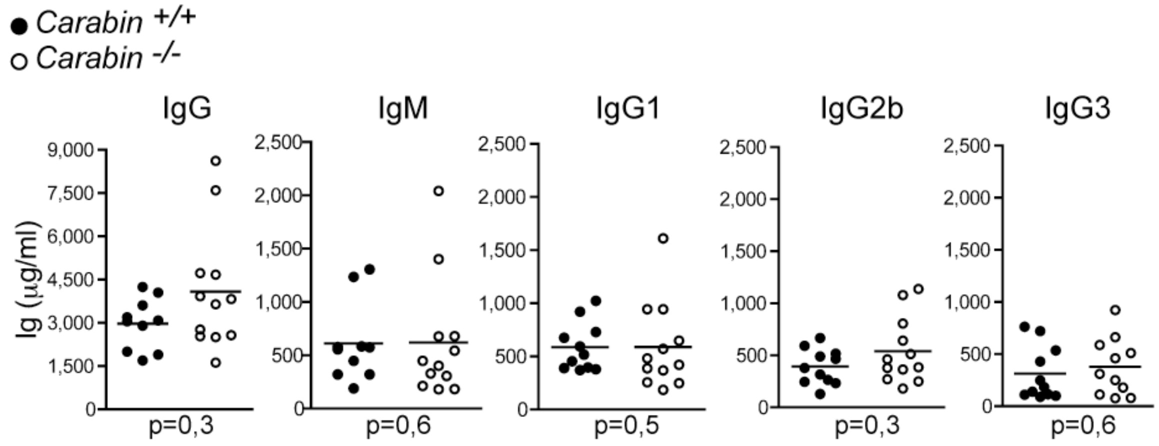

Supporting Information Figure 2. Serum Ig production in *Carabin -/-* mice. Sera from *Carabin* +/+ and *Carabin* -/- mice were collected and total IgG, IgM, IgG1, IgG2b, and IgG3 were determined by ELISA (microgram / milliliter). Each point represents the result for one animal. The results of two-tailed Mann-Whitney test are indicated.

Supporting Information Figure 3

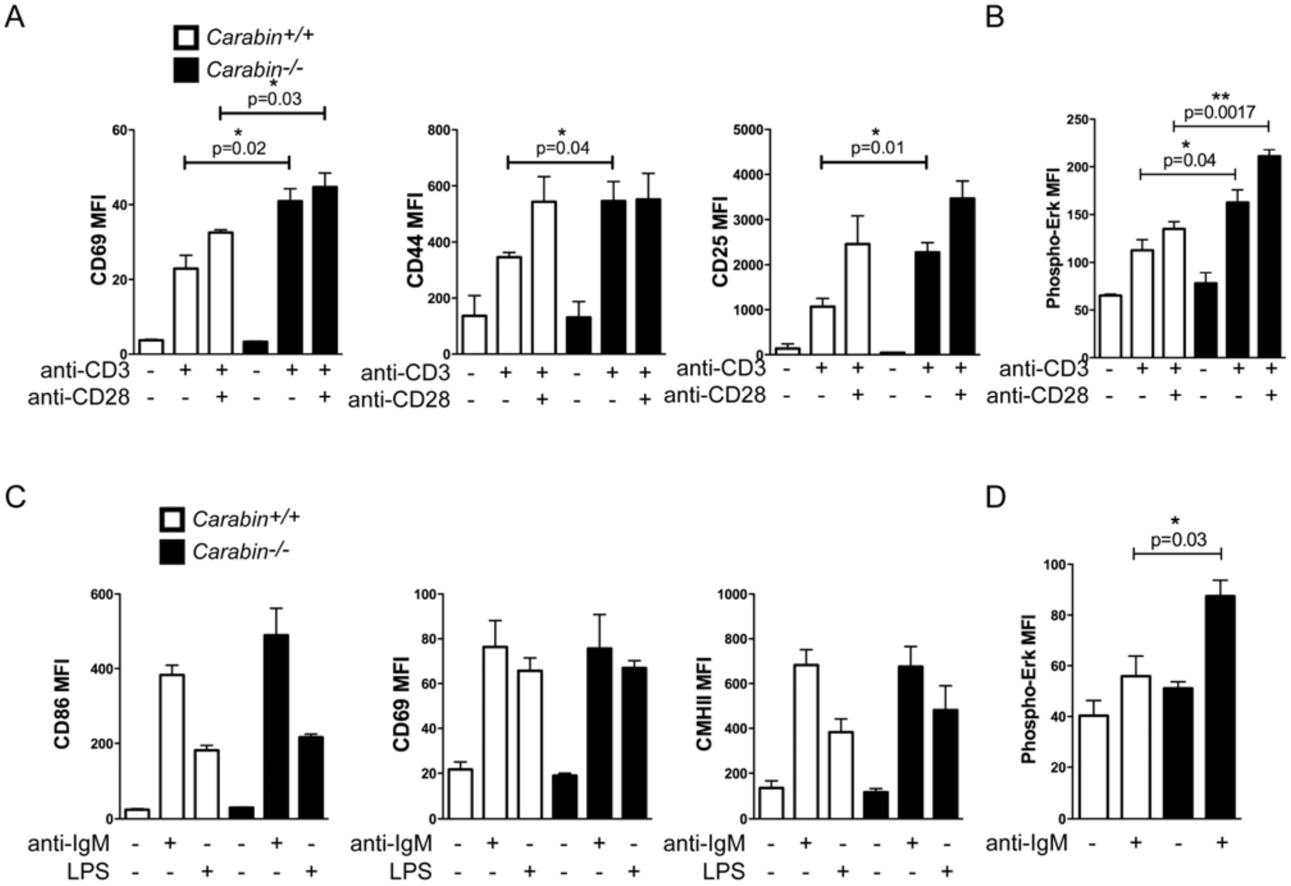

Supporting Information Figure 3. Increased response of Carabin-deficient T and B Cells. (**A**) Flow cytometry analysis of cell surface expression of CD69, CD44 and CD25 on *Carabin* +/+ and *Carabin* -/- CD4$^+$ T cells after stimulation for 72h with anti-CD3 antibody (2 µg/ml), anti-CD3+anti-CD28 antibodies (2 µg/ml each), or medium alone. (**B**) Flow cytometry analysis of Erk phosphorylation in *Carabin* +/+ and *Carabin* -/- CD4$^+$ T cells after stimulation for 10 min with anti-CD3 antibody (2 µg/ml) or with medium alone. **(C)** Flow cytometry analysis of B220+ B cell surface expression of CD86, CD69 and MHCII on *Carabin* +/+ and *Carabin* -/- CD19$^+$ B cells after stimulation for 72h with LPS (10 µg/ml) anti-IgM antibody (10 µg/ml), or medium alone and (**D**) Flow cytometry analysis of Erk phosphorylation in *Carabin* +/+ and *Carabin* -/- B220$^+$ B cells after stimulation for 10 min with anti-IgM antibody (10 µg/ml), or with medium alone. **(A-D)** Data correspond to three

independent experiments. Errors bars: standard deviation. The results of two-tailed Mann-Whitney test are indicated.

Supporting Information Figure 4

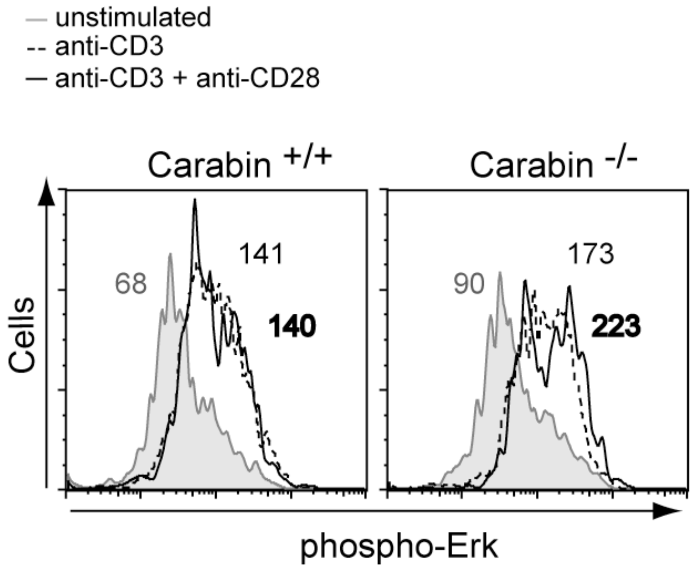

Supporting Information Figure 4. Erk phosphorylation is enhanced in *Carabin -/-* T cells after stimulation with anti-CD3 and anti-CD3 / anti-CD28 antibodies. Flow cytometry analysis of Erk phosphorylation in *Carabin* +/+ and *Carabin* -/- $CD4^+$ T cells after stimulation for 10 min with anti-CD3 antibody (2 µg/ml) (dashed line), anti-CD3+anti-CD28 antibodies (2 µg/ml each) (solid line), or with medium alone (shaded gray). Numbers indicate Mean Fluorescence Intensity. Data are representative of three independent experiments. The corresponding statistical analysis is represented in **Supporting Information Fig 3B**.

Supporting Information Figure 5

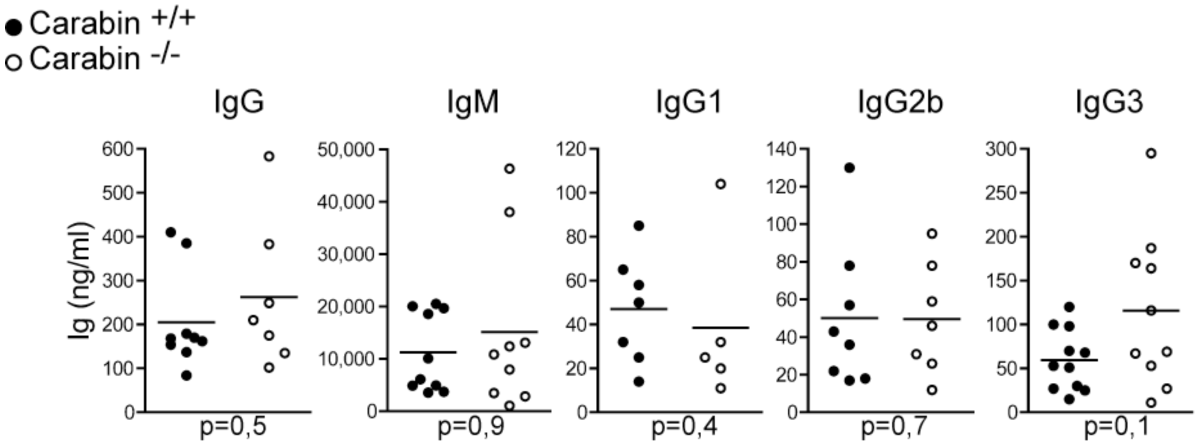

Supporting Information Figure 5. Ig levels (nanogram/milliliter) were analyzed in supernatants from splenic cell cultures, after 72h of stimulation with LPS (10 µg/ml) (in this case, IgM, IgG, IgG2b, IgG3 were quantified) or LPS (10 µg/ml) + IL-4 (20 ng/ml) (in this case, IgG1 were quantified). Each point represents the result for one animal. The results of two-tailed Mann-Whitney test are indicated.

Supporting Information Figure 6

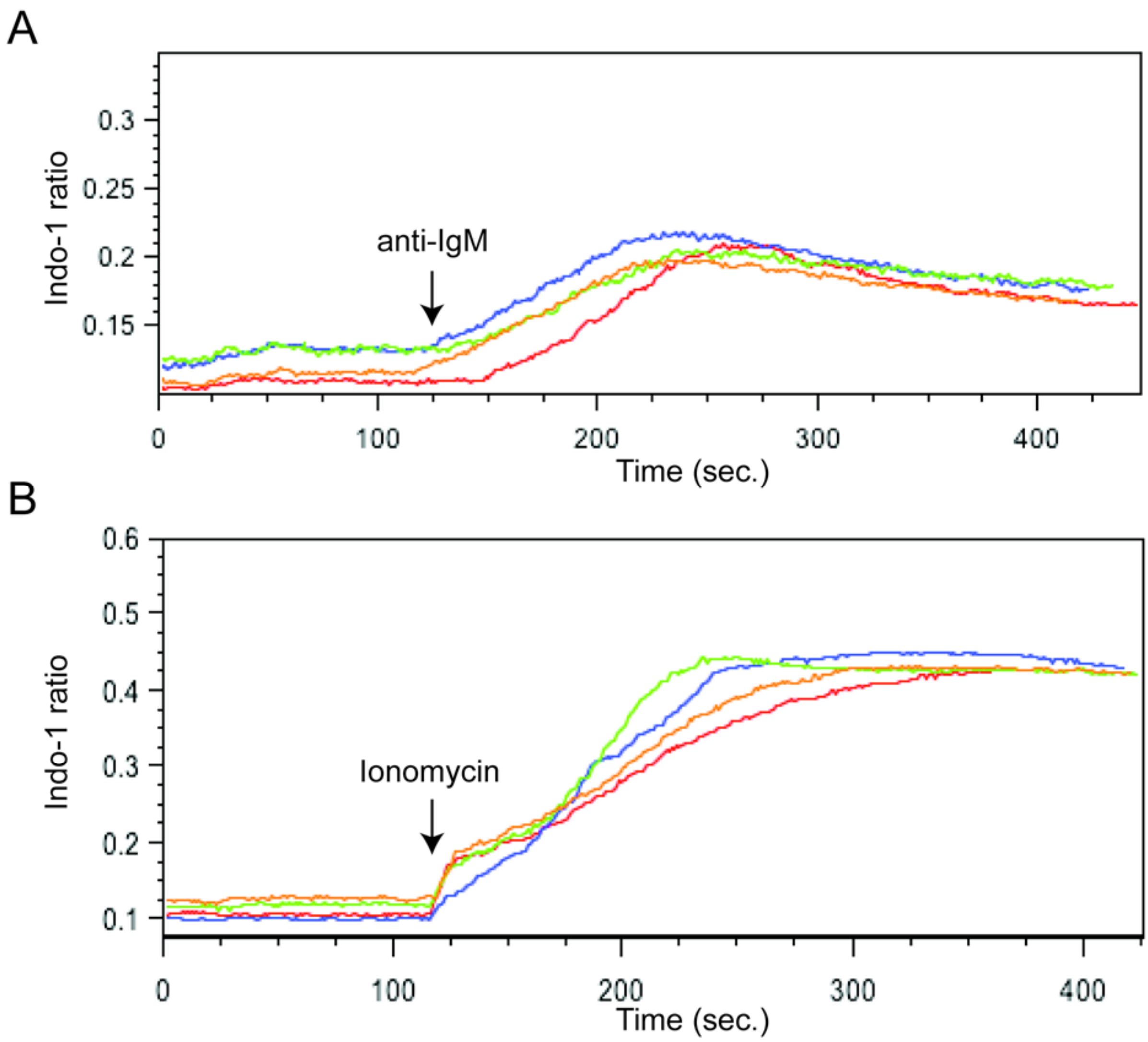

Supporting Information Figure 6. Intracellular Ca^{2+} response of Indo-1 loaded splenic purified (CD43-negative) B cells from *Carabin* +/+ (orange and red lines) and *Carabin* -/- (blue and green lines) mice. Data were collected for 120s to establish baseline violet/blue ratios; cells were then stimulated by the addition of 5 μg/ml anti-mouse IgM (**A**) or 1 μM ionomycin (**B**) and data were collected for 5 additional minutes.

Supporting Information Figure 7

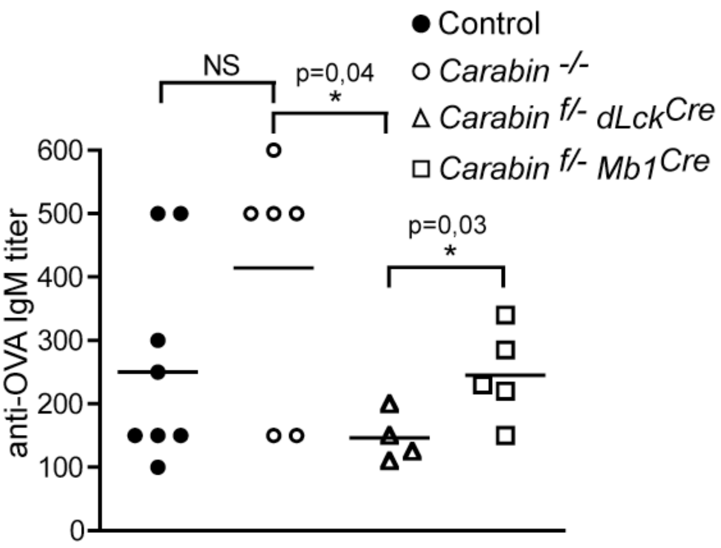

Supporting Information Figure 7. Carabin deficiency speeds up the production of anti-OVA IgM. Six- to eight-week-old mice of the indicated genotype were injected intraperitoneally with 100 µg OVA in complete Freund's adjuvant and bled seven days after injection. Anti-OVA IgM titers were determined by ELISA. Each point represents the result for one animal. The results of two-tailed Mann-Whitney test are indicated. Because the results obtained in Carabin +/+ and in Carabin f/- Cre-negative mice were not different, these animals were pooled in the same "control" group.

Supporting Information Figure 8

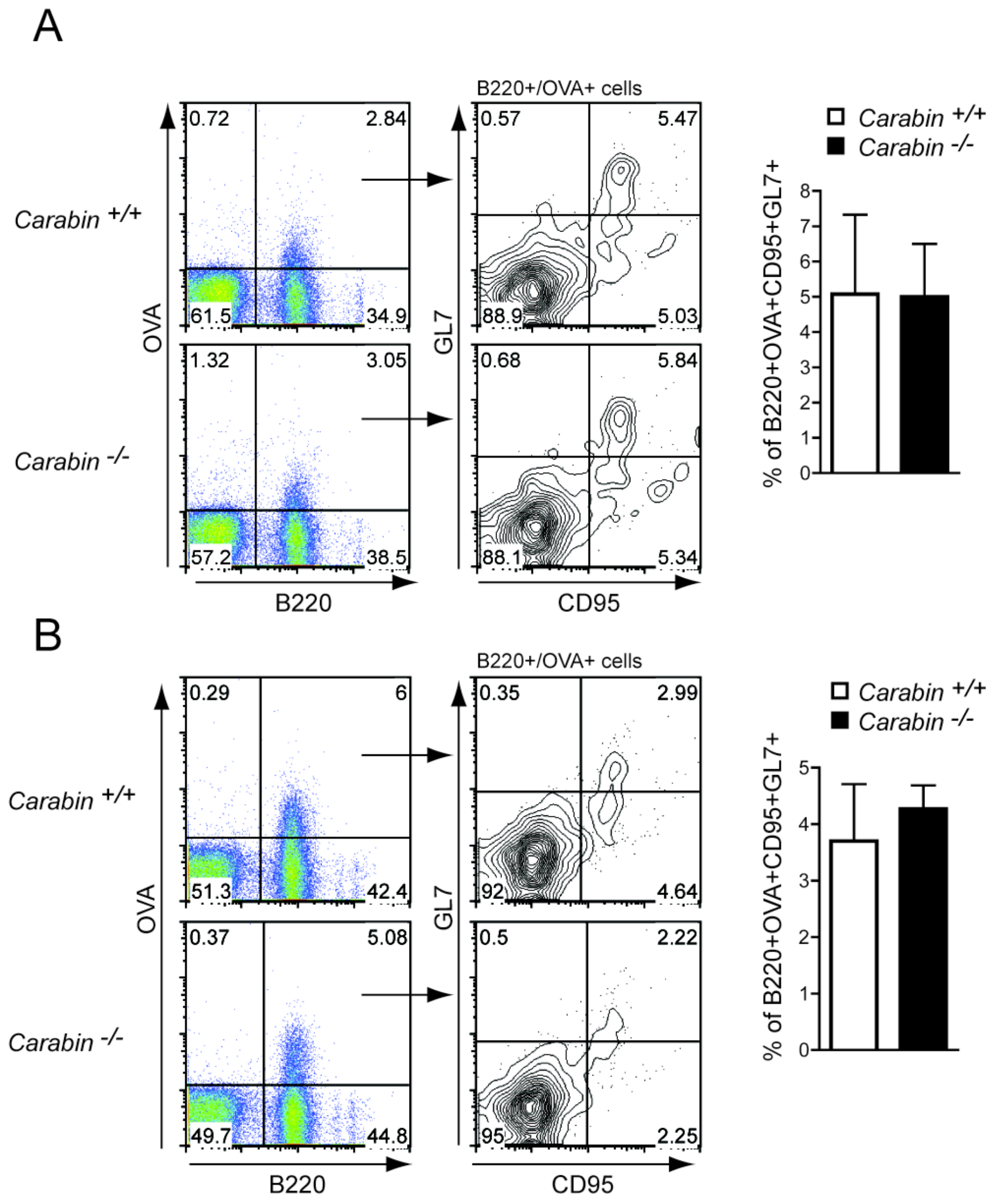

Supporting Information Figure 8. Carabin deficiency did not modify germinal center kinetics in OVA-immunized mice. (**A,B**) Flow cytometry analysis of OVA-specific B cells ($B220^+/OVA^+$) (left) and germinal center B cells ($CD95^+/GL7^+$) in OVA-specific population (middle, flow cytometry plots; right: the histograms show percentages of cells) in spleen (**A**) and lymph nodes (**B**), of Carabin -/- and Carabin +/+ mice, at day 7 after immunization with 100 µg OVA in complete Freund's adjuvant. (**A, B**) Data are representative of three independent experiments. (**A, B**; errors bars, standard deviation).

Supporting Information Figure 9

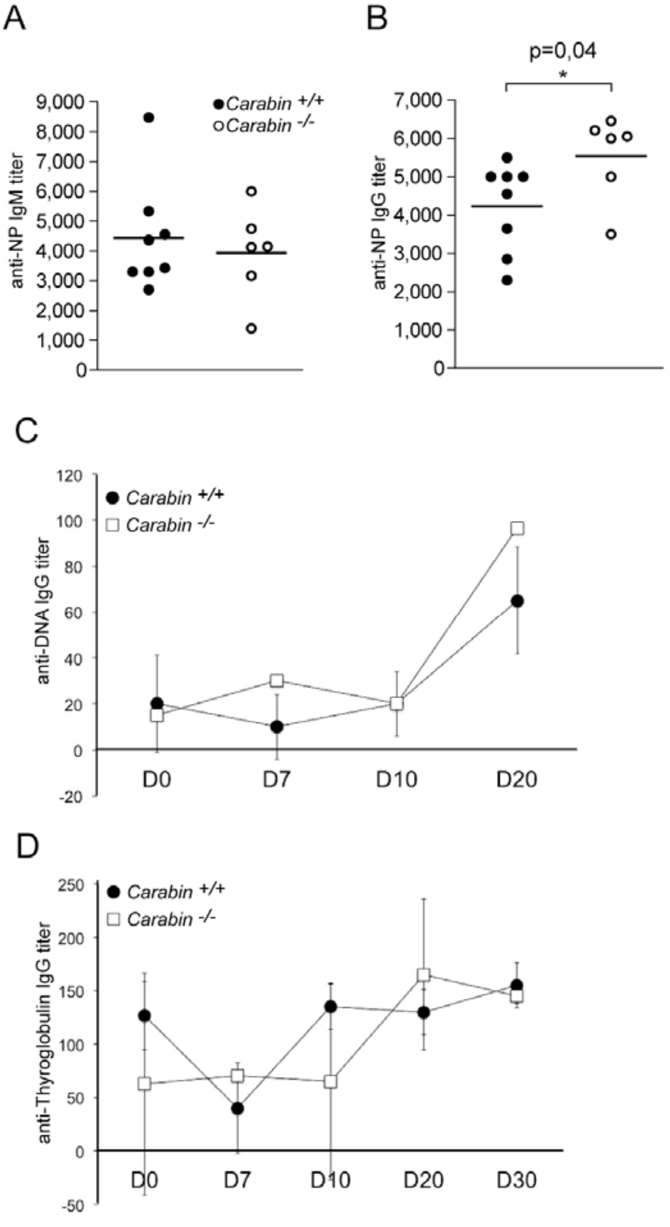

Supporting Information Figure 9. Increased early antigen-specific T-independent B cell response in Carabin KO mice. (**A, B**) 12-week-old mice of the indicated genotype were injected intraperitoneally with 100 µg NP-LPS in PBS and bled seven days after injection. Anti-NP (**A**) IgM or (**B**) IgG titers were determined by ELISA. Each point represents the result for one animal. The results of two-tailed Mann-Whitney test are indicated. (**C, D**) Anti-dsDNA **(C)** anti-thyroglobulin **(D)** IgG titers were determined by ELISA in sera of *Carabin* +/+ (n=3) and *Carabin* -/- (n=3) mice. (**C, D**; errors bars, standard deviation).

Supporting Information Figure 10

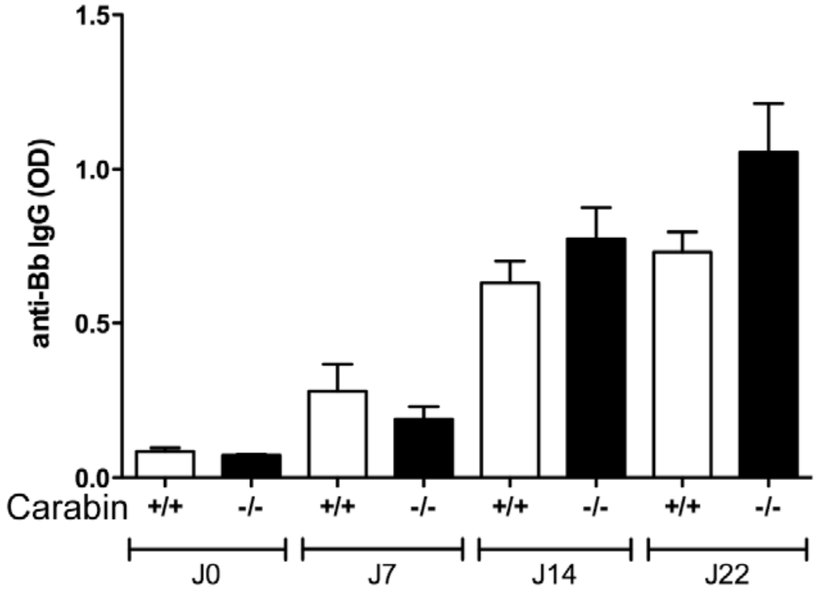

Supporting Information Figure 10. B cell response in *Carabin* deficient mice after infection with *Borrelia burgdorferi*. *Carabin* -/- (n=3) and control (n=3) mice were injected with 10^6 *Borrelia burgdorferi* (Bb) spirochetes. The anti-Bb specific IgG response was checked at the indicated times, by ELISA. The results represent the optical densities for a 1/50 serum dilution. (Errors bars, standard deviation).

Supporting Information Figure 11

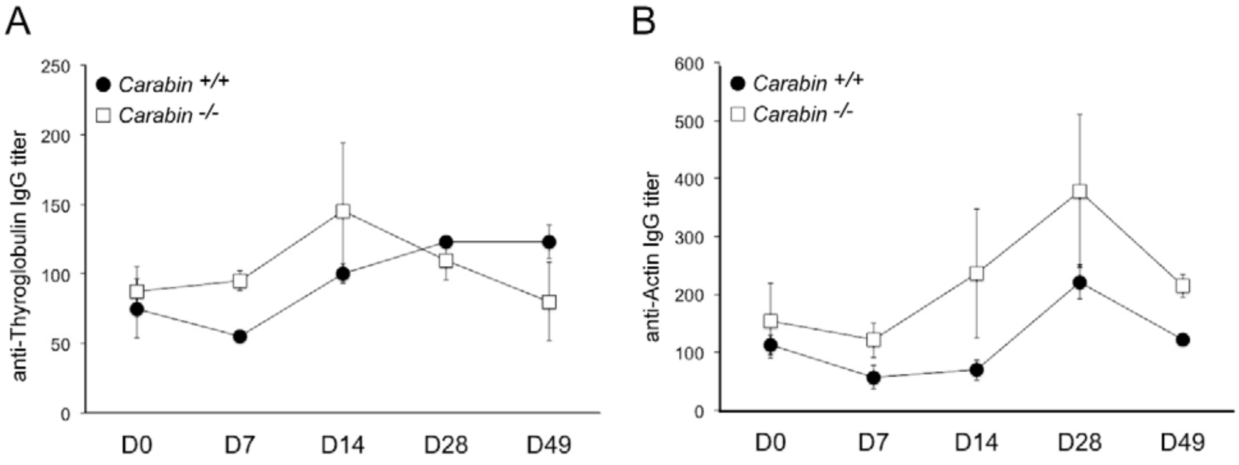

Supporting Information Figure 11. (**A, B**) Eight to ten-week-old mice of the indicated genotypes were treated with 40 μg of CpG intraperitoneally every other day for 2 weeks. Serum was collected before treatment (day 0), and at day 7, 14, 28, 49 after the first injection. Anti-thyroglobulin **(A)** and anti-actin **(B)** IgG titers were determined by ELISA (errors bars, standard deviation). **(A, B)** Data are representative of three independent experiments.

Supporting Information Figure 12

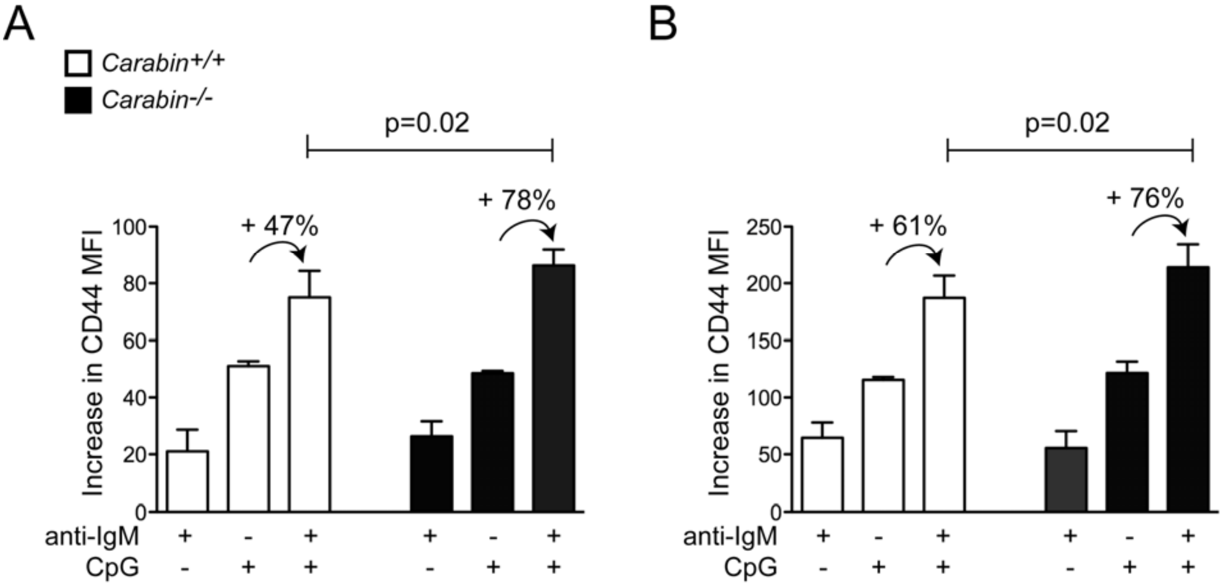

Supporting Information Figure 12. Flow cytometry analysis of cell surface expression of CD44 on *Carabin* +/+ and *Carabin* -/- splenic purified (CD43-negative) B cells after stimulation with anti-IgM (2 µg/ml), CpG-DNA (1 µg/ml), or anti-IgM (2 µg/ml) plus CpG-DNA (1 µg/ml) for 6h (**A**) and 12h (**B**) *in vitro*. Bars represent the increase of CD44 MFI on stimulated cells versus unstimulated cells (errors bars, standard deviation). Data correspond to four independent experiments. The increases of CD44 MFI on *Carabin* +/+ and *Carabin* -/- B cells were compared between anti-IgM plus CpG-DNA stimulated cells and CpG-DNA stimulated cells using a Yates' continuity corrected Chi-square test.

Supporting Information Figure 13

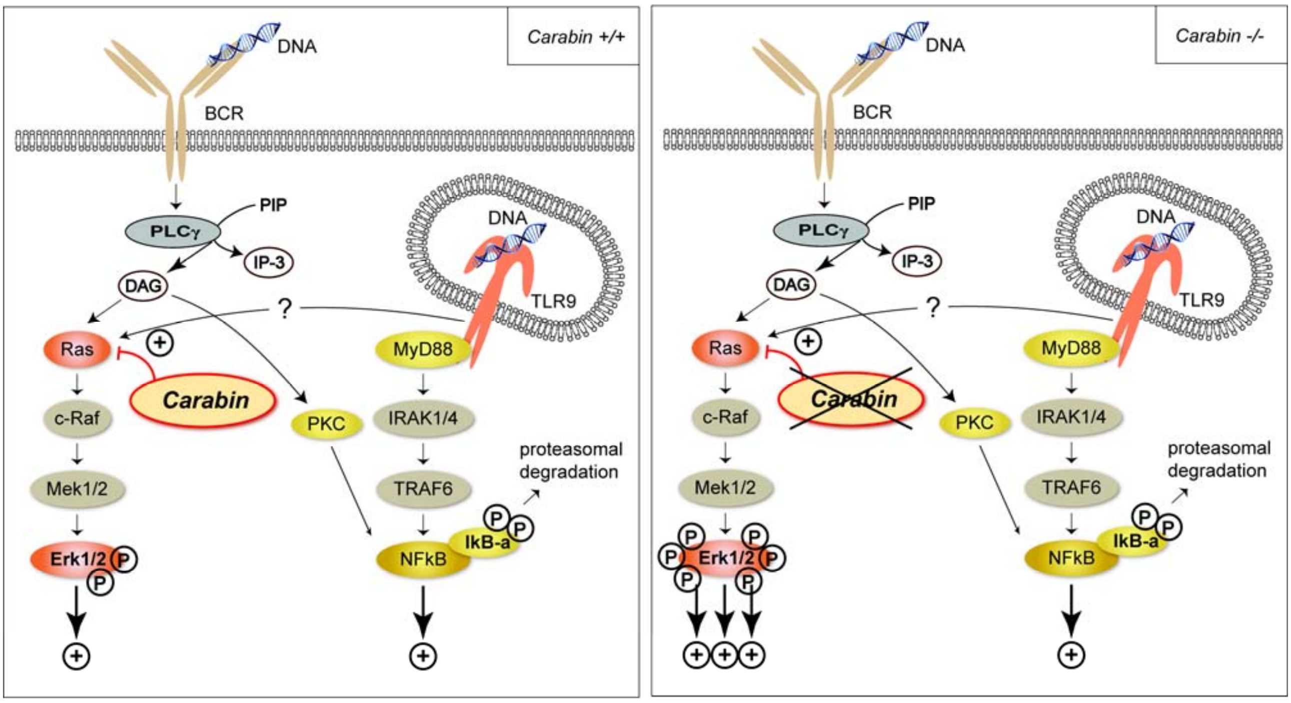

Supporting Information Figure 13. Proposed scenario of the integration of Carabin in BCR and TLR9 signaling. BCR (B-cell Receptor); TLR9 (Toll-like receptor 9); PLCγ (Phospholipase Cγ); PIP (phosphatidylinositol phosphate); DAG (Diacylglycerol); IP-3 (Inositol trisphosphate); PKC (Protein Kinase C).

	Carabin +/+	*Carabin* -/-
**Total cellularity (x10^6)**		
Spleen	112 ± 12.9	105 ± 35
Lymph nodes	4.8 ± 2.9	4.1 ± 3.5
**Spleen (% and absolute numbers)**		
B cells	50.4% ± 5.6	45.9% ± 9.3
	(48.9.10^6 ± 15.9)	(50.1.10^6 ± 12.9)
Transitional 1	15.8% ±3.1	20.3% ± 4.8
	(15.0.10^6 ± 4.7)	(21.7.10^6 ± 9.7)
Transitional 2	3.5% ±1.1	2.9% ± 0.8
	(3.3.10^6 ± 1.32)	(3.0.10^6 ± 0.6)
Follicular B cells	68.9% ± 2.29	66.1% ± 4.7
	(65.9.10^6 ± 17.6)	(68.9.10^6 ± 12.7)
Marginal zone	9.6% ± 1.1	8.2% ± 3.5
	(9.3.10^6 ± 3.2)	(9.0.10^6 ± 3.6)
CD4+ T cells	23.9% ± 8.8	21.8% ± 4.9
	(22.3.10^6 ± 6.0)	(22.0.10^6 ± 7.1)
CD8+ T cells	15.5% ± 4.3	14.4% ± 4.9
	(14.8.10^6 ± 3.7)	(14.1.10^6 ± 4.3)
**Lymph nodes (% and absolute numbers)**		
B cells	22.8% ± 5.3	27.2% ± 3.6
	(1.13.10^6 ± 0.8)	(1.0.10^6 ± 0.8)
CD4+ T cells	43.7% ± 4.6	40.2% ± 1.2
	(1.9.10^6 ± 1.2)	(1.8.10^6 ± 1.5)
CD8+ T cells	29.0% ± 4.5	29.7% ± 3.7
	(1.3.10^6 ± 0.7)	(1.3.10^6 ± 1.1)
**Bone marrow (%)**		
ProB/Pre-B	12.6% ± 3.2	15.2% ± 5.5
Immatures	4.5% ± 0.7	4.2% ± 2.5
Transitional	1.0% ± 0.5	0.9% ± 0.4
Recirculating mature	6.2% ± 2.3	5.2% ± 1.8
**Thymus (%)**		
CD4+ T cells	9.2% ± 0.5	8.4% ± 0.4
CD8+ T cells	4.3% ± 0.03	4.3% ± 0.6
DN	2.1% ± 0.2	3.1% ± 1.1
DP	84.3% ± 0.2	84.1% ± 2.2

Supporting Information Table 1. Cellularity of lymphoid organs and respective subpopulations in Carabin KO mice. Quantitation of lymphoid populations in the indicated tissues from the indicated genotypes of 12-week-old mice. The total cellularity of spleen and

lymph nodes is shown in the top panel. B cell subpopulations were identified by flow cytometry with the following markers: B cells ($B220^+IgM^+$); Pro/PreB ($B220^+IgM^-$); Immature ($B220^{med}IgM^+$); recirculating mature ($B220^{high}IgM^+$); transitional 1 ($IgM^+CD23^-CD21^-$); transitional 2 ($IgM^+CD23^+CD21^{high}$); follicular ($IgM^+CD23^+CD21^{low}$) and marginal zone ($IgM^+CD23^-CD21^{high}$). DN: double-negative; DP: double-positive T cells. Means and standard deviation of six Carabin -/- and seven Carabin +/+ mice are shown.
